# Generation of hepatic spheroids using human hepatocyte-derived liver progenitor-like cells for hepatotoxicity screening

**DOI:** 10.7150/thno.34520

**Published:** 2019-09-18

**Authors:** Zhenyu Wang, Weijian Li, Hongshu Jing, Ming Ding, Gongbo Fu, Tianjie Yuan, Weijian Huang, Mengjun Dai, Dan Tang, Min Zeng, Yi Chen, Hongdan Zhang, Xuejing Zhu, Yuan Peng, Qigen Li, Wei-Feng Yu, He-Xin Yan, Bo Zhai

**Affiliations:** 1 Department of Interventional Oncology, Renji Hospital, Jiaotong University School of Medicine, Shanghai, China;; 2 Department of Anesthesiology and Critical Care Medicine, Renji Hospital, Jiaotong University School of Medicine, Shanghai, China;; 3 International Cooperation Laboratory on Signal Transduction, Eastern Hepatobiliary Surgery Hospital, Second Military Medical University, Shanghai, China;; 4 Celliver Biotechnology Inc., Shanghai, China;; 5 Organ Transplantation Center, Changhai Hospital, Second Military Medical University, Shanghai, China.; 6 Shanghai Cancer Institute, Renji Hospital, Shanghai Jiaotong University School of Medicine, 25/Ln 2200 Xietu Road, Shanghai, 200032, China.

**Keywords:** HepLPCs, hepatocyte spheroid, heterogeneity, idiosyncratic drug-induced liver injury, TKIs.

## Abstract

**Rationale**: The idiosyncratic drug-induced liver injury (iDILI) is a major cause of acute liver injury and a key challenge in late-stage drug development. Individual heterogeneity is considered to be an essential factor of iDILI. However, few *in vitro* model can predict heterogeneity in iDILI. We have previously shown that mouse and human hepatocytes can be converted to expandable liver progenitor-like cells *in vitro* (HepLPCs). However, the limited proliferation potential of human HepLPCs confines its industrial application. Here, we reported the generation of a novel hepatocyte model not only to provide unlimited cell sources for human hepatocytes but also to establish a tool for studying iDILI *in vitro*.

**Methods**: Human primary hepatocytes were isolated by modified two-step perfusion technique. The chemical reprogramming culture condition together with gene-transfer were then used to generate the immortalized HepLPC cell lines (iHepLPCs). Growth curve, doubling time, and karyotype were analyzed to evaluate the proliferation characteristics of iHepLPCs. Modified Hepatocyte Maturation Medium and 3D spheroid culture were applied to re-differentiate iHepLPCs.

**Results**: iHepLPCs exhibited efficient expansion for at least 40 population doublings, with a stable proliferative ability. They could easily differentiate back into metabolically functional hepatocytes *in vitro* within 10 days. Furthermore, under three-dimensional culture conditions, the formed hepatic spheroids showed multiple liver functions and toxicity profiles close to those of primary human hepatocytes. Importantly, we established a hepatocyte bank by generating a specific number of such cell lines. Screening for population heterogeneity allowed us to analyze the *in vitro* heterogeneous responses to hepatotoxicity induced by molecular targeted drugs.

**Conclusions**: In light of the proliferative capacity and the heterogeneity they represented, these iHepLPCs cell lines may offer assistance in studying xenobiotic metabolism as well as liver diseases *in vitro*.

## Introduction

Liver is the largest internal organ in the human body, responsible for metabolic homeostasis. Hepatocytes perform essential liver functions, including plasma protein secretion, bile production, detoxification, metabolism, and storage of vitamins and minerals. They are the most predominant parenchymal cell type, accounting for approximately 80% of the adult liver mass 1. Human primary hepatocytes are the 'gold standard' 2 for the *in vitro* pharmaco-toxicology screening and establishment of bioartificial liver support systems 3. They are also used for research on liver disease (such as viral hepatitis) and development of chimeric liver humanized mouse models, and are of particular interest for cell-based therapies 4-6.

Consequently, the demand for human primary hepatocytes has increased considerably. Unfortunately, the use of these cells is limited by inadequate supply, high cost, and low *in vitro* proliferation capacity. Although oxygenated or microfabricated co-cultures can stabilize the metabolic functions for a few weeks, the proliferative capacity of these cells is still lost 7, 8. These constraints have prompted a large-scale search for alternative cell sources, such as hepatoma cell lines (such as HepG2 and HepaRG), stem-cell derived hepatocytes and immortalized hepatocytes. In contrast to primary cells, these cells are readily available, and usually have unlimited growth potential and high reproducibility. However, hepatoma cell lines have a single genotype, hence are not suitable for heterogeneous investigations, and their tumor background results in blunted sensitivity to toxic insults 7, 9. Given that primary hepatocytes cannot be cultured for long, this leads to inefficient immortalization and even the immortalized ones express fetal markers such as α-fetoprotein and lose multiple hepatocyte functions 10. In spite of recent progress in human pluripotent stem cells technology, the hepatocytes differentiated from these cells also express fetal markers and their metabolic activities are limited 7, 11.

Recently, we demonstrated that mouse and human hepatocytes can be converted to expandable liver progenitor-like cells *in vitro* (HepLPCs) 12, recapitulating the reversible ductal metaplasia responsible for restoration of hepatocyte mass after liver injury 13, 14. HepLPCs can be efficiently expanded and differentiated back to perform mature hepatic functions while maintaining their genome stability and integrity. However, the limited proliferation potential of human HepLPCs (about 10-15 rounds of proliferation) confines its industrial application. Here, we report an efficient method to establish multiple human immortalized HepLPCs (iHepLPCs) based on specific culture condition 12, 13. All these cell lines have similar proliferative properties and maintained individual heterogeneity. More importantly, they retained the ability to differentiate back to mature hepatocytes. The three-dimensional differentiated cells form spheroids in suspension and they perform hepatocyte functions at a level similar to that of primary hepatocytes. This system can be used in diverse applications including drug toxicity screening. Our results demonstrate that they are a suitable model for predicting toxicity and investigating the idiosyncratic drug-induced liver injury (iDILI).

## Materials and Methods

### Human Subjects

All protocols involving human tissue were reviewed and approved by the Renji Hospital of School of Medicine of Shanghai Jiaotong University institutional review boards.

### Human primary hepatocyte isolation and purification

Human primary hepatocytes were isolated by the modified two-step perfusion technique, as previously described 13. Briefly, liver tissue was perfused with pre-perfused buffer (0.5 mM EGTA (Sigma-Aldrich), 5 mg/ml BSA (Sigma-Aldrich), 1×Hanks without Ca^2+^ and Mg^2+^) for 15-30 min at 37℃ and then perfused with enzymatic dissociation buffer (1 mg/ml collagenase type IV (Sigma-Aldrich), 1×Hanks with Ca^2+^ and Mg^2+^), for 20-40 min at 37℃. The digestion was stopped by addition of cold DMEM/F12 (Gibco) + 10% v/v Fetal Bovine Serum (Gibco), 1% v/v penicillin/streptomycin/glutamine solution (Gibco). The suspension was then filtered through a 100 μm Nylon cell strainer, centrifuged at 50 g for 3 min and resuspended in 35 ml cold DMEM/F12 + 1.5 ml 10 × HBSS + 13.5 ml Percoll (GE healthcare, density 1.130 g/ml). Cells were pelleted by centrifugation at 100 g for 10 min and washed 3 times in cold DMEM/F12. In the purification step, hepatocytes were synchronously incubated with Alexa Fluor®488-conjugated anti-human CD24 antibody (Biolegend) and FITC-conjugated anti-human EpCAM antibody (Biolegend), at 4℃ for 30 min. After staining, cells were sorted by fluorescence-activated cell sorting (FACS), FITC Mouse IgG2b(Biolegend)be converted and Alexa Fluor® 488 Mouse IgG2a (Biolegend) were used as isotype controls. The viability of purified hepatocytes was determined by Trypan blue (Sigma-Aldrich).

### Establishment of the iHepLPC bank

Purified hepatocytes with about 90% viability were used for the expansion experiment. Primary hepatocytes from six donors which met vitality requirements were expanded and immortalized. Briefly, cells were plated on a Matrigel-coated culture dish and cultured in TEM as described previously 12, 13. 1-2 weeks after seeding, clonal cells were passaged at a ratio of 1:3 after dissociation with Accutase (eBioscience). Medium was changed every day. For immortalization, HepLPCs were plated in Matrigel-coated culture plates in TEM as described previously 7. After 24 h, cells were transduced with viral particles containing HPV E6/ E7 genes. The cells were cultured for an additional 72 h in TEM, then treated with 10 μg/ml puromycin (Sigma-Aldrich) twice. After puromycin selection, iHepLPCs were further expanded in TEM and cryopreserved in liquid nitrogen using LiveCyte^TM^ Cell Freezing Medium (Cryowise Medical Technology. Inc., Shanghai).

### Growth curve and doubling time

A total of 5000 cells were seeded on 6-well Matrigel-coated plates and counted on the designated days. Population doubling time was calculated using the web tool provided by http://www.doubling-time.com/compute.php.

### Karyotype analysis

The cultured iHepLPCs at passage 30 in exponential growing phase were incubated with 100 ng/mL colcemid for 40 min at 37 °C. The cultures were then washed and dissociated into single cells using Accutase and processed as described 13. Karyotype analysis was performed at the Karyotype analysis department of Beijing Biocytogen. Chromosomes from at least 40 metaphase-arrested cells were counted.

### HepaRG and HepG2 culture

Both differentiated and undifferentiated HepaRG cells were purchased from BIOPREDIC INTERNATIONAL. After thawing, the undifferentiated HepaRG cells were cultured in complete William's E medium (William's E medium (Gibco) supplemented with 10% fetal bovine serum, 1% penicillin/streptomycin/glutamine solution, 5 mg/mL insulin (Sigma-Aldrich), and 0.5 μM hydrocortisone hemisuccinate (Sigma-Aldrich)), as previously described 15. The differentiated HepaRG were maintained in HepaRG™ Maintenance/Metabolism Medium (BIOPREDIC INTERNATIONAL, France) for further hepatocyte function analysis. HepG2 cell lines were obtained from ATCC and were cultured in DMEM (Gibco) containing 10% FBS and 1% penicillin-streptomycin (Gibco).

### Monolayer differentiation

For monolayer hepatic-differentiation, iHepLPCs were seeded and kept in TEM for 2-4 days until they reached full confluence. The medium was then changed to modified Hepatocyte Maturation Medium (mHMM) 12, 13, 16 and cultured for 9-15 days for further maturation; medium was changed every day. The modified HMM (mHMM) comprised of DMEM/F12 supplemented with N2 and B27, 10 μM DAPT (TargetMol), 30 μM Dexamethasone (Sigma-Aldrich), 10 μM SB431542 (TargetMol), and 10 μm Forskolin (TargetMol). Light microscopic images were captured with Nikon Ta2-FL.

### 3D differentiation

For 3D spheroid hepatic-differentiation, 1x10^6^ iHepLPCs, undifferentiated HepaRG cells and HepG2 cells were seeded on the low-attachment 6-well plate. iHepLPCs and HepG2 cells started aggregating within 6 h and formed cellular spheres after 24 h in TEM and DMEM, respectively. The medium was then changed to mHMM, and cultured for 7-10 days for further maturation. The 3D spheroid differentiation of HepaRG cells was slightly different. 1x10^6^ HepaRG cells were seeded on low-attachment 6-well plate, the cellular spheres formed after 72 h by shaking horizontally at 30 rpm in a complete William's E medium, and then the medium was changed to mHMM for at least 14 days for further maturation. PHCs spheroid cultures were carried out as previously described 17. 1x10^6^ cells were seeded with Williams E medium containing 2 mM L-glutamine, 100 unit's/ml penicillin, 100 μg/ml streptomycin, 10 μg/ml insulin, 5.5 μg/ml transferrin, 6.7 ng/ml sodium selenite, 100 nM dexamethasone, and 10% FBS. Spontaneous self-aggregation of the hepatocytes initiated spheroid formation. From day 3 or 4 after seeding, when the spheroids were sufficiently compact, the medium was replaced with mHMM. The culture media of all cellular spheres were changed every 2-3 days.

### Quantitative real-time PCR (qPCR)

Gene expression was examined by qPCR analysis using protocols previously described 13, 15. Total RNA was extracted from the cells and 3D spheroids using Trizol reagent (Beyotime Biotechnology, China) according to the manufacturer's protocols. cDNA samples were synthesized using the GoScript™ Reverse Transcription System (Promega, USA), according to manufacturer's protocol. The concentration and purity of the RNA or cDNA were determined by ND-1000 spectrophotometer (ThermoFisher Scientific, USA). Quantitative real-time PCR was performed using SYBR Green qPCR Mix (2X, High ROX) (Beyotime Biotechnology, China) on the ABI 7300PLUS Real-Time PCR platform (Life Technologies, USA) according to manufacturer's protocol. Gene transcription was calculated using the ΔΔ^Ct^ method, normalized to the housekeeping gene 18s. Gene specific primers were synthesized by Huagen Biotech (China) and their sequences are shown in Table S1.

### RNA sequencing and bioinformatic analysis

Total RNA was isolated using RNeasy mini kit (Qiagen, Germany). The paired-end libraries were constructed using TruSeq Stranded mRNA LTSample Prep Kit (Illumina, San Diego, CA, USA) according to the manufacturer's instructions. RNA sequencing was performed on the Illumina platform (HiSeqTM 2500, Illumina, Shanghai OE Biotech. Co., Ltd.) and 150 bp paired-end reads were generated. Sequencing raw reads were preprocessed by filtering out rRNA reads, sequencing adapters, short-fragment reads and other low-quality reads using Trimmomatic 18. The Hisat2 19 was used to map the cleaned reads to the hg38 reference genome with default parameters. Read counts of each gene were summarized using HTSeq 20 and normalized using TMM (trimmed mean of M values) method. The edgeR was employed to detect differentially expressed genes (fold change above 2 with adjusted p-values below 0.05). Original data were uploaded to the Gene Expression Omnibus database (accession number GSE124528). The following transcriptomic data were included in the gene expression profile analyses: HepG2 (GSM2205676 and GSM2205677), differentiated HepaRG (GSM1834797 and GSM1834796), human primary hepatocyte (GSM2815712 and GSM2815717) and HepLPCs (GSM2815713 and GSM2815718).

### Albumin and α-1- antitrypsin production

To determine the secretion of human Albumin and α-1-antitrypsin, supernatants of cells and 3D spheroids were collected after 24 h of culture. Freshly isolated primary hepatocytes were seeded on 12-well plates and maintained in mHMM for 24 h until collection of supernatants. After collection of supernatants, all cells were lysed in RIPA buffer, and protein concentrations were measured using a BCA Protein Assay Kit (Both from Beyotime Biotechnology). These supernatants were examined using a Human Albumin ELISA kit and Human Alpha-1-Antitrypsin ELISA Kit (both from Bethyl Laboratory, UK). All data were corrected for time and total protein.

### Urea production and ammonia elimination

To determine the functionality of the cells in terms of urea production and ammonia elimination, cells and 3D spheroids were incubated in mHMM supplemented with 3 mM NH_4_Cl. Supernatants were collected 24h after NH_4_Cl induction. Freshly isolated primary hepatocytes were seeded on 12-well plates and maintained in mHMM containing 3 mM NH_4_Cl for 6 h until collection of supernatants. After collection of supernatants, all cells were lysed in RIPA Lysis and Extraction Buffer. Thereafter, protein concentrations were measured using a BCA Protein Assay Kit. Urea and NH_4_^+^ concentrations were measured using the QuantiChromTM Urea Assay Kit (BioAssy Systems, USA) and the enzymatic colorimetric assay (Megazyme International), respectively. All data were corrected for time and total protein.

### Staining and imaging

Paraffin-embedded tissue sections (3μm) were deparaffinized and rehydrated in graded alcohol concentrations. After antigen retrieval using sodium citrate buffer, sections were incubated with anti-CYP3A4, anti-CYP2D6, anti-GSTA2 and anti-MRP2 (All from proteintech, USA) and anti-NTCP (Aviva Biosystems, USA) antibodies overnight at 4^o^C. Stained tissue sections were visualized using HRP Conjugated goat anti-Rabbit IgG (H+L) secondary antibody and 3, 3-diaminobenzidine (DAB) (Both purchased from DAKO, Denmark). For monolayer culture, differentiated and undifferentiated iHepLPCs cells were cultured in 12-well plates and then fixed with 4% Paraformaldehyde. After blocking and permeabilization, cells were incubated with anti-CYP3A4 (proteintech, USA) and anti-HNF4a (Abcam, UK) antibodies overnight at 4^o^C, and then visualized using Alexa Fluor 488 Conjugated goat anti-Rabbit IgG (H+L) secondary antibody or Alexa Fluor 555 Conjugated Donkey Anti-Mouse IgG(H+L) secondary antibody and 4',6-diamidino-2-phenylindole (DAPI) (Both purchased from Beyotime Biotechnology, China). PAS staining of the monolayer culture was carried using the Glycogen Stain kit (Nanjing Jiancheng Bioengineering Institute), following the instructions provided by the manufacturer. Images were captured with Nikon Ta2-FL.

### Functional Polarization Assay

Differentiated and undifferentiated iHepLPCs cultured on 12-well plates were incubated for 20 min with 5 μM 5(6)-carboxy-2', 7'-dichlorofluorescein diacetate (CDCFDA). Cell cultures were subsequently washed with ice-cold phosphate-buffered saline containing calcium and magnesium and then imaged with Nikon Ta2-FL fluorescence microscope.

### CYP450 Induction

To evaluate CYP450 induction, cultured cells were incubated with 25 μM of rifampicin or 50 μM of omeprazole dissolved in culture medium for 72 h. CYP450 gene expression was quantified by qPCR.

### CYP450 activity analysis

To test CYP450 activity, the 3D differentiated iHepLPCs cells and freshly isolated primary hepatocytes were cultured in the medium with the following substrates at different concentrations (phenacetin/CYP1A2, bupropion/CYP2B6, diclofenac/CYP2C9, testosterone/CYP3A4, 7-hydroxycoumarin/UGT) in 200 μl incubation medium for 60 min at 37℃ (conducted in RILD Biotech., Shanghai). Finally, 800 μl cold methanol was added to stop the reaction followed by centrifugation. Cell supernatants were collected for measurement of CYP450 activity by LC-MS/MS (Agilent 1200 HLPC and ABI 4000 mass spectrometer). Total cell protein concentration was used to normalize the data.

### Assessment of Cellular Toxicity

For cellular toxicity assessment, we used the 3D differentiation cells on the Corning® 384-well Spheroid Microplates (Figure S1). For acute toxicity assay, compounds were dissolved in DMSO or mHMM depending on its solubility to prepare a series of solutions with increasing concentrations. These compounds include Acetaminophen (30000, 7500, 1875, 469, 117, 29, 7 or 0 μM), Amiodarone (128, 32, 8, 2, 0.5, 0.125, 0.032 or 0 μM), Chlorpromazine (2048, 512, 128, 32, 8, 2, 0.5 or 0 μM), Diclofenac (1600, 400, 100, 25, 6.25, 1.56, 0.4 or 0 μM), Troglitazone (500, 125, 31, 7.8, 2, 0.5 or 0 μM), Mannitol (6400, 3200, 1600, 800, 400, 200 or 100 μM), Erlotinib, Cabozantinib, Foretinib, Gefitinib (250, 64, 16, 4, 1, 0.25 or 0 μM), Crizotinib (100, 25, 6.25, 1.56, 0.4, 0.1, 0.25 or 0 μM), Ceritinib (280, 70, 17.5, 4, 1, 0.5, 0.06 or 0 μM), Tepotinib (200, 50, 12.5, 3.2, 0.8, 0.2, 0.05 or 0μM), and Lapatinib (400, 100, 25, 6.25, 1.56, 0.4, 0.1 or 0 μM). Cellular spheres were exposed to these increasing concentrations of compounds on day 9. The viability of cells was quantified using PrestoBlue™ Cell Viability Reagent (Invitrogen, USA) after 48 h of exposure. For long-term toxicity assay, cellular spheres were exposed to increasing concentrations of fialuridine (0, 0.1, 0.33, 1, 3.3, 10, 33 or 100 μM) for 2 days, 7 days, 14 days, and 28 days, after which the viability of the cells was determined using PrestoBlue™ Cell Viability Reagent (Invitrogen, USA). Normalized Dose-dependent toxicity curves and TC_50_ values were plotted using GraphPad Prism software (GraphPad Software Inc, USA).

### Toxicological Endpoint Assays (Apoptosis, Cholestasis, Steatosis)

Toxicological endpoints were evaluated for five hepatotoxic compounds at 25% toxic concentration (TC_25_) values identified from experiments above after 48 h of exposure. Apoptosis was determined by the DeadEnd terminal deoxynucleotidyl transferase mediated dUTP nick end labeling (TUNEL; Beyotime, China) according to manufacturer's instructions. Apoptotic nuclei were normalized to total DAPI (Beyotime, China)-stained nuclei. Intracellular lipids were quantified by Nile Red (Solarbio, China) according to manufacturer instructions. Staining intensity was normalized to that of negative control. Hepatic cholestasis was quantified using CDCFDA staining as described above and was normalized to the number of Hoechst-stained nuclei.

### Statistical analysis

All statistical analyses were performed using GraphPad Prism 7. The two-tailed unpaired t-test was used to compare statistical significance between two mean values. The one-way ANOVA was used with Dunnett correction for multiple comparisons of multiple values to a single value or Tukey correction for multiple comparisons of multiple values to each other. A p-value < 0.05 was considered statistically significant.

## Results

### iHepLPCs from different donors exhibited similar morphology and proliferation characteristics

**Figure 1:**
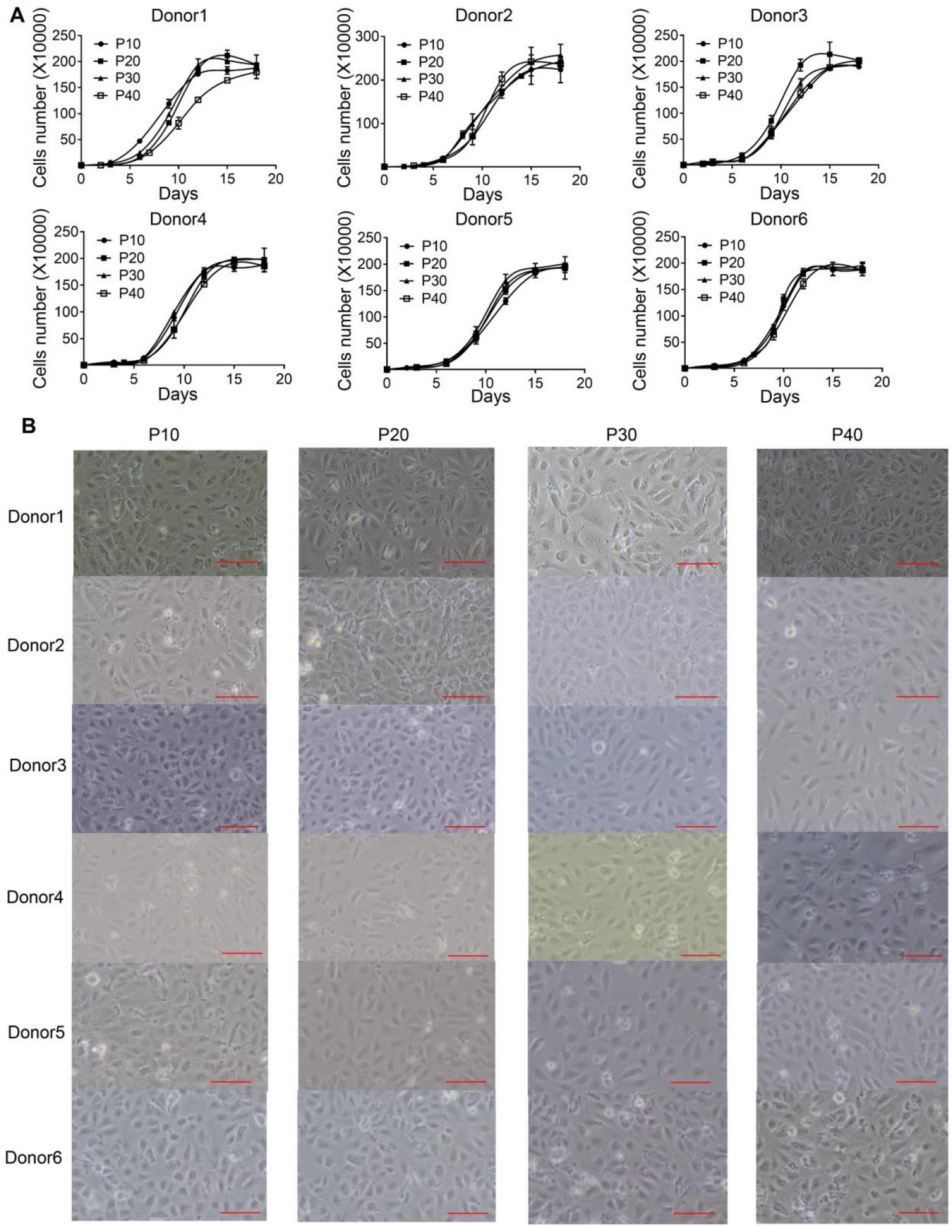
** iHepLPCs derived from different donors have similar morphology and proliferation characteristics. (A).** Growth curve of different generations of iHepLPCs from six donors. All iHepLPCs underwent active division and the mean population doubling time was 26-32 h. **(B)** Images of different generations of iHepLPCs from donor 1 - 6. No obvious morphological changes were found. The scale bar is 100 μm.

We previously identified a transition and expansion medium (TEM) that allows for the conversion of human hepatocytes into progenitor cells (LPC) *in vitro* 13. However, the limited proliferation potential of these cells restricts its industrial application. To address this issue, we developed an efficient protocol to establish multiple immortalized hepatocyte-derived liver progenitor-like cells (iHepLPCs). Six iHepLPCs from six different donors were generated using HPV E6/E7 overexpression by culturing in TEM 7, 12, 13(Figure S1). All iHepLPCs underwent active division, and the mean population doubling time was between 26-32 h (Table S2). They displayed stable proliferation characteristics and morphology across different generations (Figure 1A-B, Table S2). Karyotypic analyses of all six iHepLPCs at passage 30 showed that donor 1, 2, 3, 6- derived cells maintained normal diploid karyotypes, but the others partly exhibited aneuploidy and chromosomal translocation (Figure S2). Although aneuploidy is one of the characteristics of normal mature hepatocytes 21, chromosomal translocation seems to be caused by long-term culturing. Whole-genome transcriptome analysis of HepLPCs before and after immortalization showed that E6/E7 overexpression caused transcriptional changes in a number of genes related to cell cycle, apoptosis and DNA repair (Figure S3A-B). For example, the transcription of 28 proliferation-related genes was higher in iHepLPCs compared with HepLPCs, (Figure S3C).

### iHepLPCs differentiated back in the modified hepatic maturation medium

**Figure 2:**
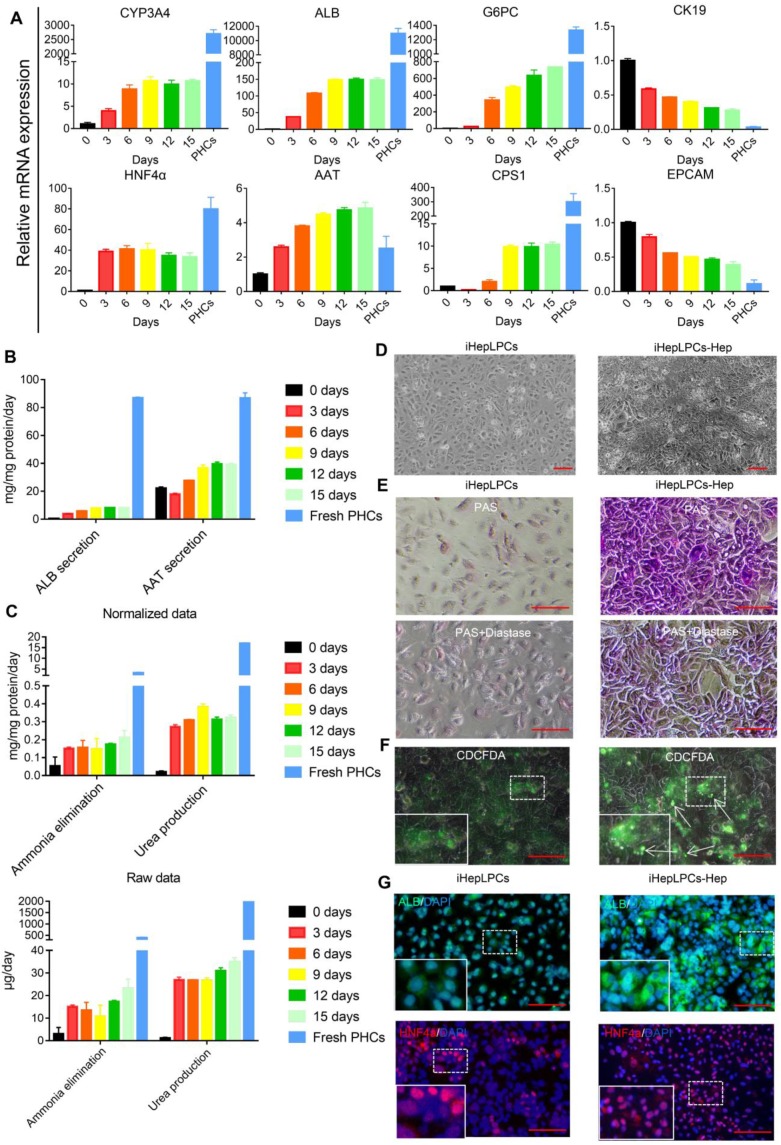
** iHepLPCs converted back in the hepatic maturation medium. (A)** qPCR analyses for the expression of CYP3A4, ALB, AAT, G6PC, CPS1, HNF4a, CK19 and EpCAM during hepatic-differentiation of iHepLPCs in mHMM (passage 30) from day 0 to day 15. Error bars represent s.d.; n = 3. **(B)** Albumin secretion, AAT secretion, **(C)** urea production and ammonia elimination after 24 h of culture, measured from supernatants. Error bars represent s.d.; n = 3 **(D)** Images of iHepLPCs before (left) and after (right) 9 days of differentiation. iHepLPCs-Hep underwent distinct morphological changes. The scale bar is 100 μm. **(E)** PAS staining with or without diastase and **(F)** CDCFDA staining of iHepLPCs before (left) and after (right) 9 days of differentiation. arrows indicated functional bile canaliculi, the scale bar is 100 μm. **(G)** Immunofluorescence staining of albumin, HNF4a in iHepLPCs before (left) and after (right) 9 days of differentiation, the scale bar is 100 μm.

Next, we evaluated the hepatic differentiation ability of iHepLPC using the modified hepatic maturation medium (mHMM) 12, 13, 16. After a 15-days culture, hepatocyte-like cells differentiated from iHepLPCs (iHepLPCs-Hep) at passage 30 showed a gradual increase in the expression of drug metabolism enzyme CYP3A4, albumin, α-antitrypsin (AAT), urea cycle key enzyme CPS1 and gluconeogenesis enzyme G6PC.These increases stabilized around day 9 (Figure 2A-C). On the other hand, the expression of precursor cell markers CK19 and EpCAM showed a gradual decrease (Figure 2A). The heat map of RNA sequencing (RNA-Seq) data showed that mHMM significantly changed the gene expression pattern of iHepLPCs-Hep relative to iHepLPCs (Figure S4A); including significant up-regulation of liver function genes and down-regulation of cell cycle genes (Figure S4B). Compared to undifferentiated cells, iHepLPCs-Hep exhibited polygonal morphology with increased ratio of cytoplasm: nucleus (Figure 2D). Periodic acid Schiff (PAS) reaction and CDCFDA staining showed that iHepLPCs-Hep accumulated glycogen and formed bile canaliculi (Figure 2E-F). Consistent with qPCR results, immunofluorescence staining showed that the re-differentiated cells expressed a higher amount of albumin and HNF4α than the undifferentiated iHepLPCs (Figure 2G). However, iHepLPCs-Hep lacked key mature hepatocyte functions and required further functional enhancement (Figure 2A-C).

### 3D spherical differentiation promoted further hepatic maturation

**Figure 3:**
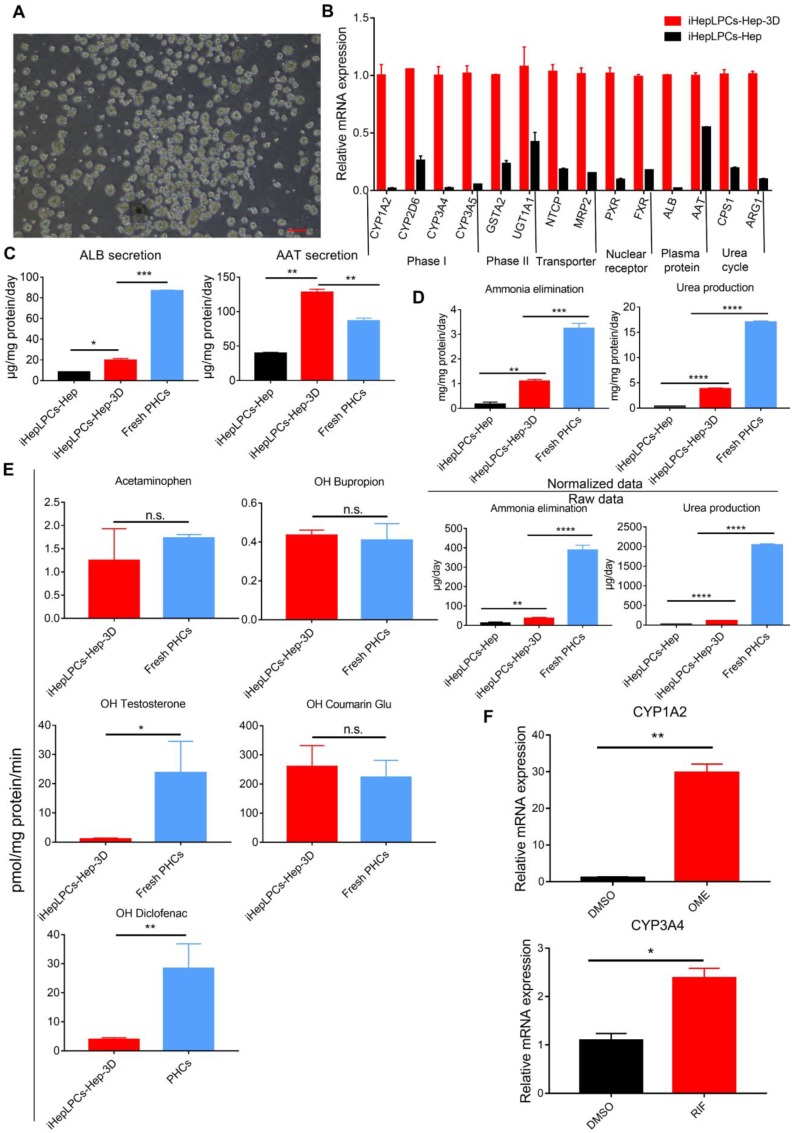
** 3D spherical differentiation promoted further hepatic maturation. (A)** Images of iHepLPCs-Hep-3D; the scale bar is 200 μm. **(B)** qPCR analysis of iHepLPCs-Hep-3D and iHepLPCs-Hep showed that 3D spherical differentiation significantly increased the expression of liver-specific genes. Error bars represent s.d.; n = 3 **(C)** Comparison of levels of albumin, antitrypsin, **(D)** urea production and ammonia elimination between freshly isolated PHCs, iHepLPCs-Hep and iHepLPCs-Hep-3D. Error bars represent s.d.*P < 0.05, **P < 0.01, ***P<0.001, ****P<0.0001; n = 3. **(E)** Metabolic activities of CYPs in 3D-HepLPCs-Hep, PHCs (freshly isolated). The metabolic products of Acetaminophen, OH-Bupropion, OH-Diclofenac, OH-Testosterone and OH-Coumarin Glu were determined by liquid chromatography-tandem mass spectrometry according to standard curves. Error bars represent s.d.; n = 3. **(F)** Induction of CYP450 activity in iHepLPCs-Hep-3D in response to 72-h stimulation with omeprazole or rifampicin. Omeprazole significantly induced CYP1A2 expression, whereas rifampicin induced CYP3A4 expression. Error bars represent s.d.; *P < 0.05, **P < 0.01; n = 3.

There is growing evidence that 3D culture can dramatically enhance the degree of hepatic functions 11, 13, 22, 23. To promote further maturation of iHepLPCs-Hep, we generated 3D cell spheroids from the iHepLPCs, then differentiated these spheroids using mHMM (iHepLPCs-Hep-3D, Figure 3A). We used the Live/Dead staining to analyze the viability of the interior cells during the cultivation. The results showed that the majority of cells were viable on the 9th day. Only a few of dead cells were randomly present in the spheroids, even in those which diameter was greater than 200 μm (Figure S5). qPCR analyses showed that 3D culture increased the expression of several genes associated with liver function, including phase I drug metabolizing enzyme CYP1A2, CYP2D6, CYP3A4, CYP3A5; phase II drug metabolizing enzyme GSTA2, UGT1A1; nuclear factor PXR, FXR; plasma protein albumin, α-antitrypsin and urea cycle related genes CPS1 and ARG1 compared to the expression in monolayer culture (Figure 3B). ELISA assays showed that albumin secretion (3.5-fold) and α-antitrypsin secretion (4-fold) were upregulated significantly in iHepLPCs-Hep-3D compared to the expression in 2D monolayer culture, which were 0.25 and 1.5 fold higher than those of freshly isolated primary hepatocytes, respectively (Figure 3C). iHepLPCs-Hep-3D also produced much higher level of urea and eliminated ammonia more efficiently than the 2D culture and other cell lines commonly used in bioartificial liver (Figure 3D, Figure S6). Furthermore, the iHepLPCs-Hep-3D were able to metabolize Acetaminophen, OH-Bupropion, OH-Diclofenac, OH-Testosterone and OH-Coumarin Glu (Figure 3E) efficiently. Importantly, the activity of CYP450 in iHepLPCs-Hep-3D was increased by classical agonists. Omeprazole upregulated CYP1A2 expression by 29.8±1.6-fold (over DMSO-treated control). Rifampicin upregulated CYP3A4 expression by 2.4±0.14-fold (over DMSO-treated control, Figure 3F).

**Figure 4:**
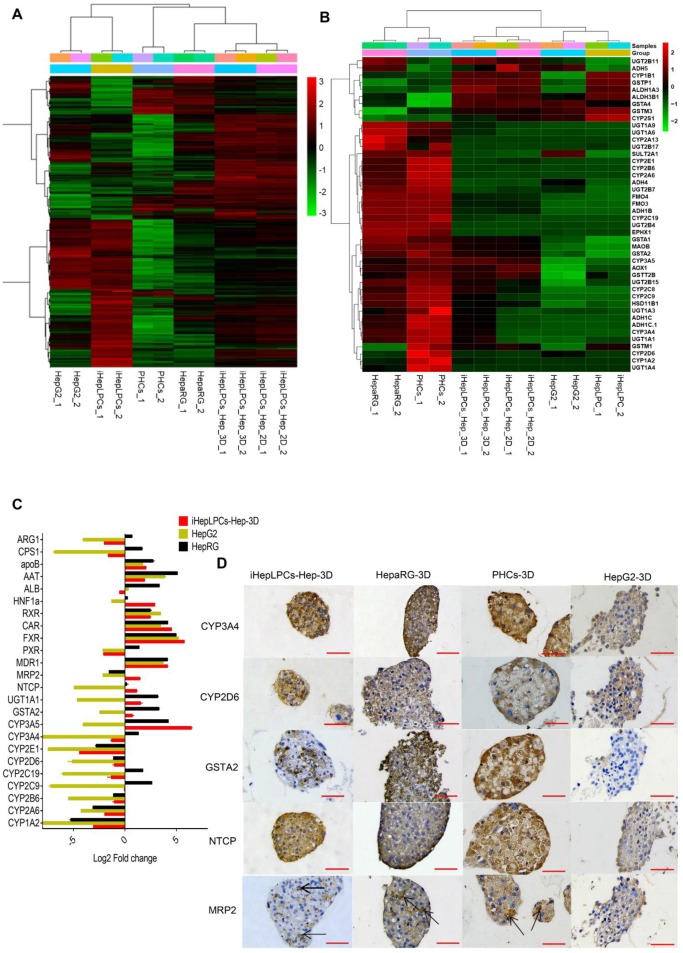
** Transcriptomics and immunohistochemical analysis of iHepLPCs-Hep-3D. (A)** Euclidean hierarchical clustering of HepG2, HepaRG, iHepLPCs, iHepLPCs-Hep, iHepLPCs-Hep-3D and PHCs using differentially expressed genes (≥2-fold changes and P < 0.001) in iHepLPCs and iHepLPCs-Hep-3D. **(B)** Euclidean hierarchical clustering of the expression of main drug metabolizing genes in HepG2, HepaRG, iHepLPCs, iHepLPCs-Hep, iHepLPCs-Hep-3D and PHCs. Each element represents log2 (normalized expression), as scaled by the corresponding color legends from 2 repeats. **(C)** Analysis of log2 fold changes in expression levels of 24 liver function genes in HepG2, HepaRG, and iHepLPCs-Hep-3D cells relative to PHCs, measured by qRT-PCR. **(D)** Immunohistochemical analyses of CYP3A4, CYP2D6, GSTA2, NTCP and MRP2 in iHepLPCs-Hep-3D, HepaRG-3D, hepG2-3D cells and PHCs-3D. The scale bar is 50 μm.

To further characterize iHepLPCs-Hep-3D, we compared gene expression profiles of iHepLPCs, iHepLPCs-Hep, iHepLPCs-Hep-3D with primary human hepatocytes (PHCs), hepatic hepatoma cell lines (HepG2) and differentiated HepaRG cell line (HepaRG). RNA sequencing revealed that the expression profiles of iHepLPCs-Hep-3D clustered with those of primary hepatocytes and HepaRG (Figure 4A), especially in drug metabolizing genes (Figure 4B). Similar to RNA-seq, qPCR analyses for the expression of 24 liver function-related genes in iHepLPCs-Hep-3D, HepaRG, HepG2, and PHCs confirmed that iHepLPCs-Hep-3D were much closer to PHCs and HepaRG than HepG2 (Figure 4C).

### Application of iHepLPCs-Hep-3D for *in vitro* toxicological screening

*In vitro* drug screening is the most common industrial application of human hepatocytes. We therefore investigated whether iHepLPCs-Hep-3D could effectively predict the toxicity of hepatotoxic drugs. We compared the expression of 107 drug metabolizing enzymes and transport (DMET) genes in HepaRG, HepG2, PHCs versus iHepLPCs-Hep-3D using volcano plot (Figure S7). A more uniform distribution was observed in the volcano plot that compared iHepLPCs-Hep-3D with PHCs or HepaRG, demonstrating less divergence of gene expression and therefore a similar gene expression profile in PHCs and HepaRG. Immunohistochemical staining of phase I enzymes CYP3A4, CYP2D6, phase II enzyme GSTA2, and transporter MRP2, NTCP (also is the hepatitis B virus receptor) showed that similar to PHCs-3D, both iHepLPCs-Hep-3D and HepaRG-3D, but not HepG2-3D, could effectively synthesize drug-metabolizing enzymes (Figure 4D). These results demonstrated the potential of iHepLPCs-Hep-3D for drug screening applications.

**Figure 5:**
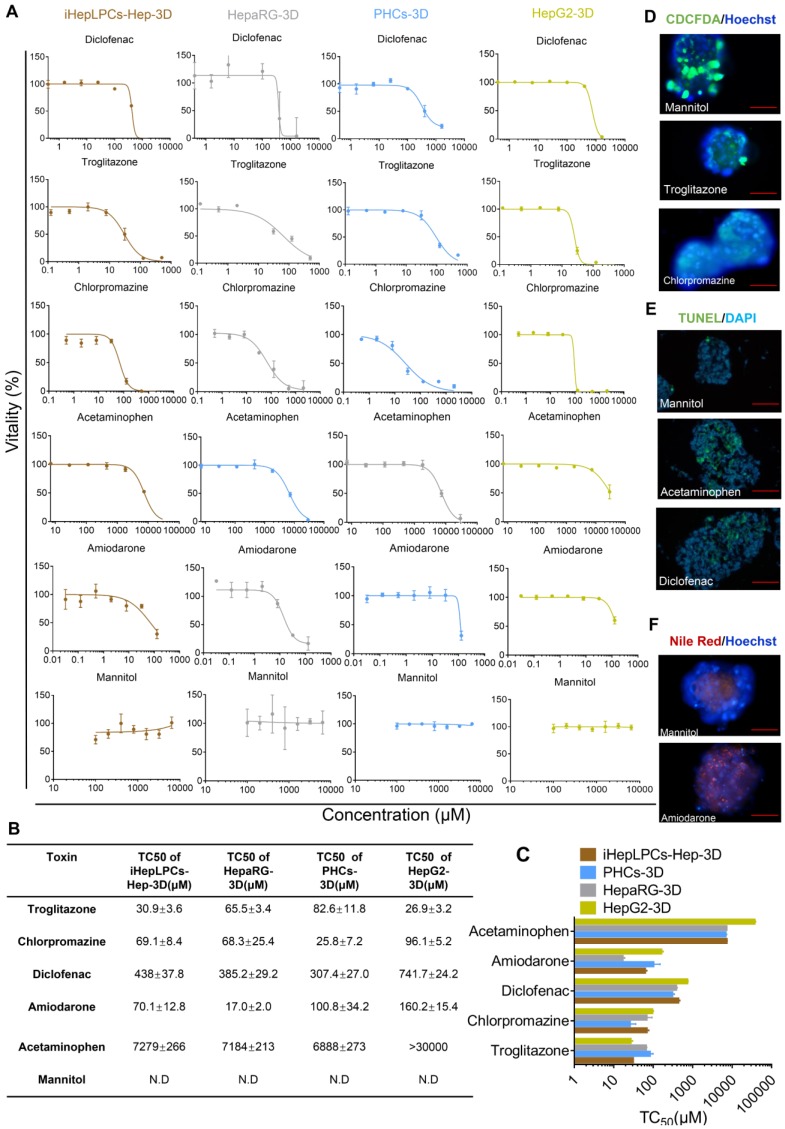
** iHepLPCs-Hep-3D can predict toxicological outcomes. (A)** Dose-dependent toxicity curves of different compounds obtained from 48-h dose responses in iHepLPCs-Hep-3D, HepaRG-3D, hepG2-3D and PHCs-3D. **(B-C)** TC_50_ values of different compounds obtained from 48-h dose responses in iHepLPCs-Hep-3D, HepaRG-3D, hepG2-3D and PHCs-3D. Error bars represent s.d.; n = 3. (D-F) Fluorescence analysis of adverse outcome pathway in iHepLPCs-Hep-3D. Loss of bile acid production (cholestasis) evaluated by CDFDA staining, lipid accumulation (steatosis) by Nile Red staining and apoptosis by TUNEL labeling of nuclei. (D) Cholestasis in iHepLPCs-Hep-3D exposed to Troglitazone for 48 h, Chlorpromazine or Mannitol (negative control). **(E)** Apoptosis of differentiated hepatocytes following 48 h of exposure to Acetaminophen, Diclofenac or Mannitol. (F) Steatosis in iHepLPCs-Hep-3D after 48 h of exposure to Amiodarone or Mannitol. All error bars indicate ± s.d. All scale bar is 50 μm.

Next, we tested five compounds that display different toxicological endpoints (e.g., cholestasis) and mannitol, which is generally regarded to be safe, in iHepLPCs-Hep-3D, HepaRG-3D, PHCs-3D, and HepG2-3D. Cells, cultured in Corning® 384-well Spheroid Microplates (Figure S1), were exposed to increasing concentrations of these compounds for 9 days, and viability was determined after 48 hours of exposure. Results are summarized as dose-dependent toxicity curves (Figure 5A) and TC_50_ as a concentration causing 50% cell death (Figure 5B). Dose-dependent toxicity curves showed a classical sigmoidal response characteristic of toxic metabolites. Importantly, a normalized TC_50_ toxicity profile generated for iHepLPCs-Hep-3D and HepaRG-3D was not significantly different from that of PHCs-3D (p=0.147 and p=0.249, respectively, n=3), whereas HepG2-3D profile was significantly different (P<0.001; n=3; Figure 5C). To explore the ability of iHepLPCs-Hep-3D to predict the toxicological response, we evaluated toxicological endpoints of the five hepatotoxic compounds defined above at TC_25_ concentrations (Figure 5D-F, Figure S8A-C). Cholestasis was evaluated by CDCFDA staining. After 48 h of exposure, cultures treated with troglitazone or chlorpromazine showed a 9- to 25-fold decrease in the number of CDF-positive bile canaliculi, compared to control (P=0.0009; n=3; Figure 5D, Figure S8A). Apoptosis was evaluated using the TUNEL assay. After 48 h of exposure, cultures treated with acetaminophen or diclofenac showed a 4- to 7-fold increase in the percent of apoptotic nuclei compared to control (P=0.0108; n=3; Figure 5E, Figure S8B). Steatosis was evaluated using Nile red lipid staining. After 48 h of exposure, cultures treated with amiodarone showed a 10-fold increase in intracellular lipids compared to control (P =0.0008; n=3; Figure 5F, Figure S8C).

To test the response of iHepLPCs-Hep-3D to chronic toxicants, we examined the chronic toxicity (4 weeks) of fialuridine which caused 5 deaths out of 15 patients in Phase II clinical trial 24. The results showed that fialuridine produced a time- and dose-dependent toxic response, and exerted a more distinct toxic effect in iHepLPCs-Hep-3D than in HepaRG-3D and HepG2-3D (Figure S9).

iDILI is a major cause of acute liver injury and a key challenge in late stage drug-development. Individual heterogeneity is considered to be an important factor of iDILI 25, 26. However, except for iPSC-induced hepatocytes 9, no effective model can predict heterogeneity in iDILI 27. To investigate whether iHepLPCs-Hep-3D might be an effective tool for studying heterogeneity, we tested the toxic effects of five iHepLPCs-Hep-3D from different donors. Dose-dependent toxicity curves showed that all six iHepLPCs-Hep-3D could predict the toxicity (Figure S10A). More importantly, TC_50_ values indicated that there was significant individual heterogeneity among the iHepLPCs-Hep-3D from different donors (Figure S10B). This heterogeneity was also confirmed by qPCR analyses (Figure S11).

### iHepLPCs-Hep-3D from different donors can predict idiosyncratic events of molecular targeted drugs

**Figure 6:**
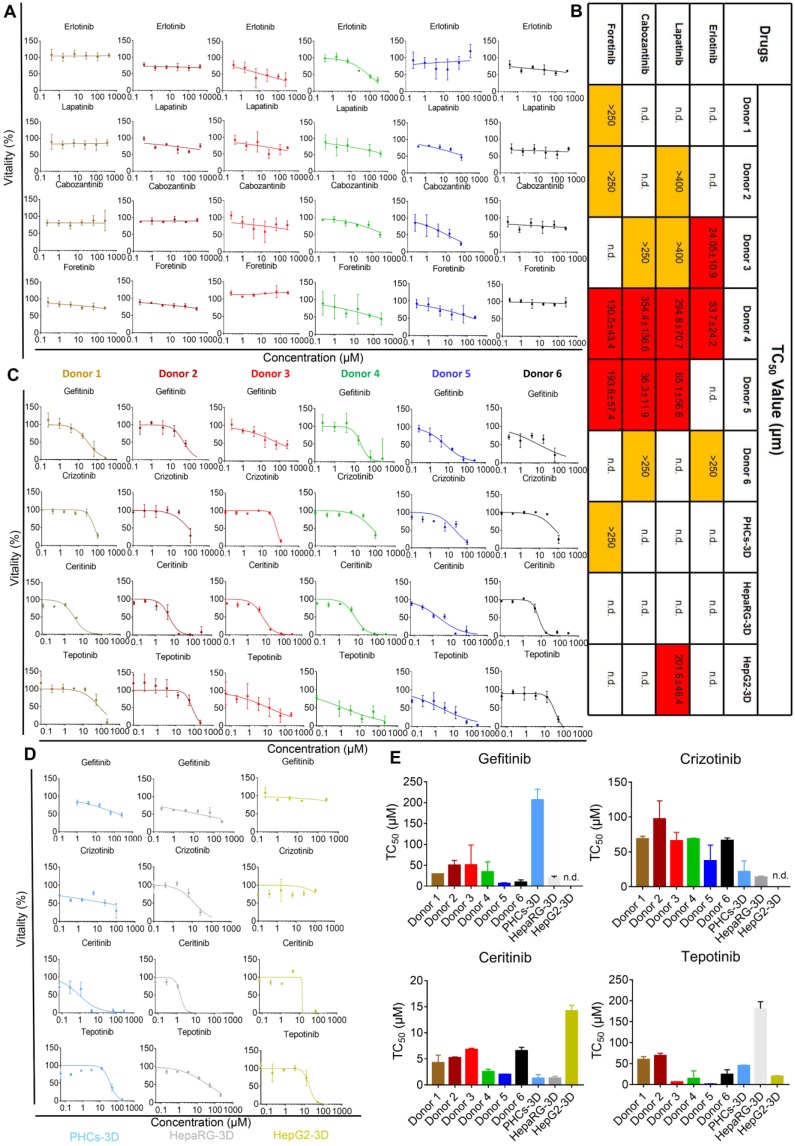
** iHepLPC-Heps-3D derived from different donors can predict idiosyncratic events of molecular targeted drugs. (A)** Dose-dependent toxicity curves of indicated drugs obtained from 48-h dose responses in iHepLPCs-Hep-3D derived from different donors, showing significant idiosyncratic events. **(B)** TC_50_ values of Erlotinib, Lapatinib, Cabozantinib and Foretinib obtained from 48-h dose response in iHepLPCs-Hep-3D derived from different donors, HepaRG-3D, hepG2-3D and PHCs-3D. Different colors represent idiosyncratic events, red elements represent more than half of the cell death and the corresponding TC_50_ values; yellow elements indicate mild toxicity of the drugs but did not cause more than half of the cell death and the TC_50_ values exceed the highest concentration tested. **(C)** Dose-dependent toxicity curves of Gefitinib, Crizotinib, Ceritinib and Tepotinib obtained from 48-h dose response in iHepLPCs-Hep-3D derived from different donors. The four drugs exhibited inherent toxicity. **(D)** The inherent toxicity of the drugs was also confirmed by PHCs-3D, HepaRG-3D, but not HepG2-3D. Their TC_50_ values are summarized in **(E)**. All error bars represent s.d.; n=3; n.d. represent no toxicity was found.

One of the main treatments for neoplastic diseases is chemotherapy, which uses molecular targeted drugs. Antineoplastic treatments are associated with several types of organ damage, such as liver damage 28. These toxicities may reflect individual genetic predispositions that impact drug metabolism, disposition, immune responses, and end-organ responsiveness 29. To investigate the potential of iHepLPCs-Hep-3D to predict iDILI caused by molecular targeting drugs, we selected eight targeted drugs (all of which are receptor tyrosine kinase inhibitors, TKIs, Table S3), which have shown hepatotoxicity in clinical trials 28, 30, 31. Dose-dependent toxicity curves showed that iHepLPCs-Hep-3D from different donors displayed significantly different toxic responses to four drugs; Erlotinib, Lapatinib, Cabozantinib, and Foretinib (Figure 6A). Lapatinib, Cabozantinib, and Foretinib showed significant toxicity to iHepLPCs-Hep-3D derived from donor 4 and donor 5, while Erlotinib displayed significant toxicity to donor 3 and donor 4. In addition, Erlotinib exerted mild toxicity on donor 6; Lapatinib exerted mild toxicity on donor 2 and donor 3; Cabozantinib exerted a mild toxicity on donor 3 and donor 6; whereas Foretinib had similar effects on donor 1 and donor 2 (Figure 6B). Interestingly, donor 4 showed a strong toxic response to all four drugs, suggesting an unknown mechanism that renders them more sensitive to TKIs (Figure 6B). In addition, except for Lapatinib (showing a significant toxicity on HepG2-3D) and Foretinib (eliciting a slight toxic reaction on PHCs-3D), no toxic reaction was found on HepaRG-3D, HepG2-3D and PHCs-3D (Figure 6B, Figure S12). These results suggest that heterogeneity may influence the toxicity of the four TKIs. According to the dose-dependent toxicity curves (Figure 6C), all the other four TKIs, Gefitinib, Crizotinib, Ceritinib and Tepotinib, showed strong toxicity to all iHepLPCs-Hep-3D, indicating that their toxicity effects were less affected by individual heterogeneity. Similar toxic effects occurred on PHCs-3D and HepaRG-3D, but not HepG2-3D (Figure 6D-E). These results indicate that iHepLPCs-Hep-3D may be an effective *in vitro* hepatocyte model to explore idiosyncratic drug-induced liver injury and predict intrinsic hepatotoxicity of new drugs.

## Discussion

Human hepatocytes are routinely applied in hepatotoxicity testing, drug metabolism, chimeric mice and are used in cell therapy to correct genetic defects or support patients with liver-assist devices 7 The reversible metaplasia of hepatocyte to biliary-like progenitor cells has been observed *in vivo* 14 and has enabled us to generate culturable progenitor cells from hepatocytes with defined chemical factors 12, 13. Furthermore, these cells can differentiate back into functional hepatocytes *in vitro*. However, the limited proliferative potential of HepLPCs 13 restricts their industrial application. Here, we describe a highly efficient method to establish immortalized HepLPC cell lines on the basis of chemical reprogramming culture conditions combined with HPV E6/E7 overexpression (Figure S1). Technically, the efficiency of generating iHepLPC cell lines was nearly 100%. These cells actively proliferated for at least 40 generations with similar doubling time (Figure 1A, Figure S2) and showed a consistent morphology (Figure 1B). These results indicate that iHepLPCs hold the potential to be a reliable and unlimited source of human hepatocytes (Table S4).

Similar to HepLPCs, iHepLPCs retained the ability to differentiate back into mature hepatocytes *in vitro*. The performance of liver functions exhibited by the cells gradually increased, stabilizing within 10 days after monolayer differentiation (Figure 2A-C), a duration that is significantly shorter than that of HepaRG differentiation (4 weeks 32, 33) and hepatic differentiation of iPSC/ESC (2-4weeks 11, 34). More importantly, when cultured in suspension, they preferably formed spheroids and displayed enhanced liver-specific functions, especially that of urea cycle and drug metabolism (Figure 3, Figure 4, Figure S6). We also found that primary hepatocytes exhibited a higher capacity to produce urea and eliminate ammonia as well as a higher expression level of drug metabolism genes. This phenomenon may be explained by the possibility that the current system is unable to effectively induce maturation of iHepLPCs. Co-culture with non-parenchymal cells, use of advanced three-dimensional culture system, and establishment of a rational combination of small molecules are needed to address this challenge in the future. Extensive analysis of gene expression profiles demonstrated that the differentiated cells clustered closely with primary hepatocytes and differentiated HepaRG cells, especially in genes related to liver function (Figure 4), demonstrating a high potential for *in vitro* application.

Adverse drug reactions (ADRs) are common health problems associated with some medications. ADR is a major issue in the pharmaceutical industry as it is responsible for attrition of drugs during development and withdrawal of drugs post-licensing 35. Among ADRs, drug-induced liver injury (DILI) is the most frequent reason for the withdrawal of approved drugs from the market, and it accounts for more than 50% of cases of acute liver failure 35, 36. In contrast to intrinsic toxicity, idiosyncratic toxicity occurs in some people, and is hard to predict 35. Although it is rare, the majority of post-marketing restrictions by the FDA are due to idiosyncratic events 26. Several studies have shown that TKIs targeting c-MET and EGFR may be effective therapeutic drugs for advanced tumors 30, 37, 38. However, it should be noted that approximately 11-50% of TKI can cause drug-induced liver injury 29. TKIs have unique toxicity profiles 29. Both on- and off-target toxicities may be due to high drug concentrations or altered end-organ sensitivity, which in turn can be a consequence of genetic polymorphisms controlling metabolism or tissue responsiveness 29, 31. In this study, iHepLPCs-Hep-3D successfully detected the hepatotoxicity of six known toxic compounds (Figure 5) and was able to demonstrate the differences in toxicity in various cells (Figure S10). For the first time, to our knowledge, we have successfully predicted the heterogeneous toxicity of several TKIs (Figure 6). These results suggest that iHepLPCs-Hep-3D derived from different donors may be a powerful tool for predicting idiosyncratic events, which will greatly reduce the incidence of ADRs.

## Conclusion

We developed a highly efficient method for establishing iHepLPCs and its differentiation scheme. *In vitro* drug screening experiments were performed to demonstrate its potential for industrial application. The results show that the iHepLPCs are likely been a powerful tool not only for the accurate and efficient drug development, but also for advanced hepatocyte-based clinical application including in bioartificial liver support device.

## Supplementary Material

Supplementary figures and tables.

## Abbreviations

AAT: α-antitrypsin
ARG1: arginase 1
ApoB: apolipoprotein B
CYP1A2: cytochrome P450 family 1 subfamily A member 2
CYP2A6: cytochrome P450 family 2 subfamily A member 6
CYP2B6: cytochrome P450 family 2 subfamily B member 6
CYP2C9: cytochrome P450 family 2 subfamily C member 9
CYP2C19: cytochrome P450 family 2 subfamily C member 19
CYP2D6: cytochrome P450 family 2 subfamily C member 6
CYP2E1: cytochrome P450 family 2 subfamily E member 1
CYP3A4: cytochrome P450 family 3 subfamily A member 4
CYP3A5: cytochrome P450 family 3 subfamily A member 5
CPS1: carbamoyl-phosphate synthase 1
CDCFDA: 5(6)-carboxy-2': 7'-dichlorofluorescein diacetate
DAPI: 4':6-diamidino-2-phenylindole
DMET: drug metabolizing enzymes and transport
DMSO: dimethyl sulfoxide
DILI: drug-induced liver injury
ELISA: enzyme-linked immunosorbent assay
FXR: farnesoid X receptor
GSTA2: glutathione S-transferase A2
HMM: Hepatocyte Maturation Medium
HNF1a: hepatocyte nuclear factor 1-alpha
HNF4a: hepatocyte nuclear factor 4-alpha
HepLPCs: liver progenitor-like cells
iHepLPCs: Immortalized HepLPCs
iHepLPCs-Hep: hepatocyte-like cells differentiated from iHepLPCs
iHepLPCs-Hep-3D: 3D cell spheroids of iHepLPCs-Hep
iDILI: idiosyncratic drug-induced liver injury
MDR1: multidrug resistance protein 1
mHMM: modified HMM
MRP2: multidrug resistance-associated protein 2
NTCP: Na1-taurocholate cotransporting polypeptide
OME: omeprazole
PXR: pregnane X receptor
PHCs: human primary hepatocytes
QPCR: quantitative real-time PCR
RIF: rifampicin
RXR: retinoid X receptor
RNA-Seq: RNA sequencing
TEM: transition and expansion medium
TKIs: tyrosine kinase inhibitors
TC_50_: 50% toxic concentration
TC_25_: 25% toxic concentration
TUNEL: terminal deoxynucleotidyl transferase mediated dUTP nick end labeling
UGT1A1: UDP glucuronosyltransferase family 1 member A1
