# Supplementary material for: Generation of hepatic spheroids using human hepatocyte-derived liver progenitor-like cells for hepatotoxicity screening
Source: Theranostics. 2019 Sep 18;9(22):6690–705. doi: 10.7150/thno.34520 (PMC6771233; doi:10.7150/thno.34520)
Supplement: Supplementary file 1 — Supplementary figures and tables. [file thnov09p6690s1.pdf]

Generation of hepatic spheroids using human hepatocyte-derived liver progenitor-like cells for hepatotoxicity screening

Zhenyu Wang[1,*], Weijian Li[1,*], Hongshu Jing[1,*], Ming Ding[1], Gongbo Fu[3], Tianjie Yuan[2], Weijian Huang[3], Mengjun Dai[1], Dan Tang[2], Min Zeng[4], Yi Chen[2], Hongdan Zhang[4], Yuan Peng[1], Qigen Li[5], Wei-Feng Yu[2], He-Xin Yan[1,2,6], Bo Zhai[1]

1. Department of Interventional Oncology, Renji Hospital, Jiaotong University School of Medicine, Shanghai, China;
2. Department of Anesthesiology and Critical Care Medicine, Renji Hospital, Jiaotong University School of Medicine, Shanghai, China;
3. International Cooperation Laboratory on Signal Transduction, Eastern Hepatobiliary Surgery Hospital, Second Military Medical University, Shanghai, China;
4. Celliver Biotechnology Inc., Shanghai, China;
5. Organ Transplantation Center, Changhai Hosipital, Second Military Medical University, Shanghai, China.
6. Shanghai Cancer Institute, Renji Hospital, Shanghai Jiaotong University School of Medicine, 25/Ln 2200 Xietu Road, Shanghai, 200032, China.

* These authors contributed equally to this work.

Corresponding authors:

Bo Zhai, Ph.D., Department of Interventional Oncology, Renji Hospital, Shanghai, 200120, China. E-mail: zhaiboshi@sina.com.

He-Xin Yan, Ph.D., Department of Interventional Oncology, Renji Hospital, Shanghai, 200120, China. E-mail: hexinyw@163.com; yanhexin@renji.com.

Wei-Feng Yu, Ph.D., Department of Anesthesiology and Critical Care Medicine, Renji Hospital, Jiaotong University School of Medicine, Shanghai, China. E-mail: ywf808@yeah.net.

Supplementary figures and figure legends

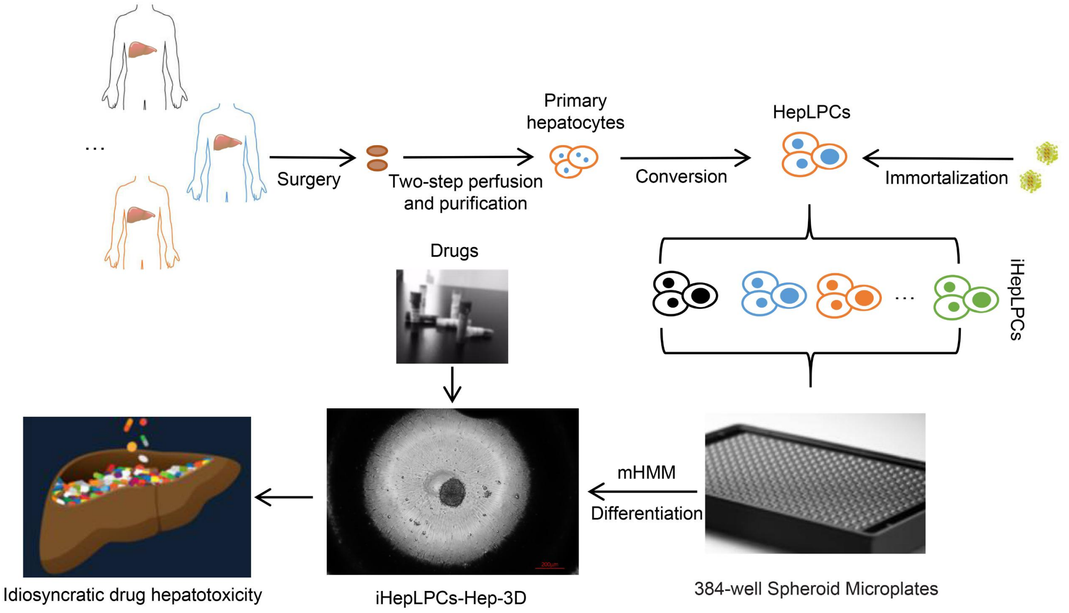

Figure S1: Experimental scheme for this research. Six iHepLPCs were generated from six donors, and then iHepLPCs-Hep-3D from different donors were established to investigate the idiosyncratic hepatotoxicity.

Donor 1

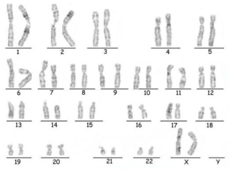

	Abnormal karyotype	Normal diploid karyotypes
Cell numbers	0	45
Percentag e	0%	100%

Donor 2

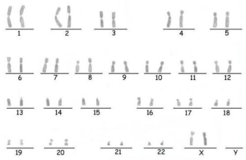

	Abnormal karyotype	Normal diploid karyotypes
Cell numbers	0	29
Percentag e	0%	100%

Donor 3

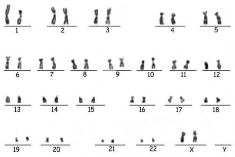

	Abnormal karyotype	Normal diploid karyotypes
Cell numbers	0	34
Percentag e	0%	100%

Donor 4

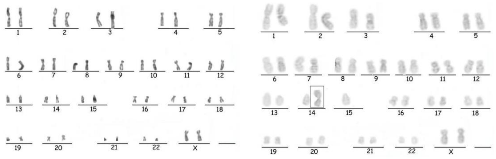

	2n=45, rob(14,15)	Normal diploid karyotypes
Cell numbers	12	22
Percentag e	35%	65%

Donor 5

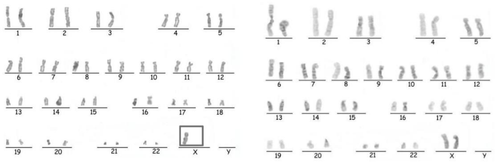

	2n = 45	Normal diploid karyotypes
Cell numbers	9	24
Percentag e	27%	73%

Donor 6

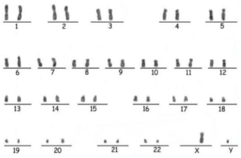

	Abnormal karyotype	Normal diploid karyotypes
Cell numbers	0	33
Percentag e	0%	100%

Figure S2: Karyotype analysis of all six iHepLPCs at passage 30. Donor 1, 2, 3, 6-derived cells had normal diploid karyotypes. The chromosome Robertsonian translocation was found in 35% of cells derived from donor 4, and aneuploidy was found in 27% of the cells derived from donor 5.

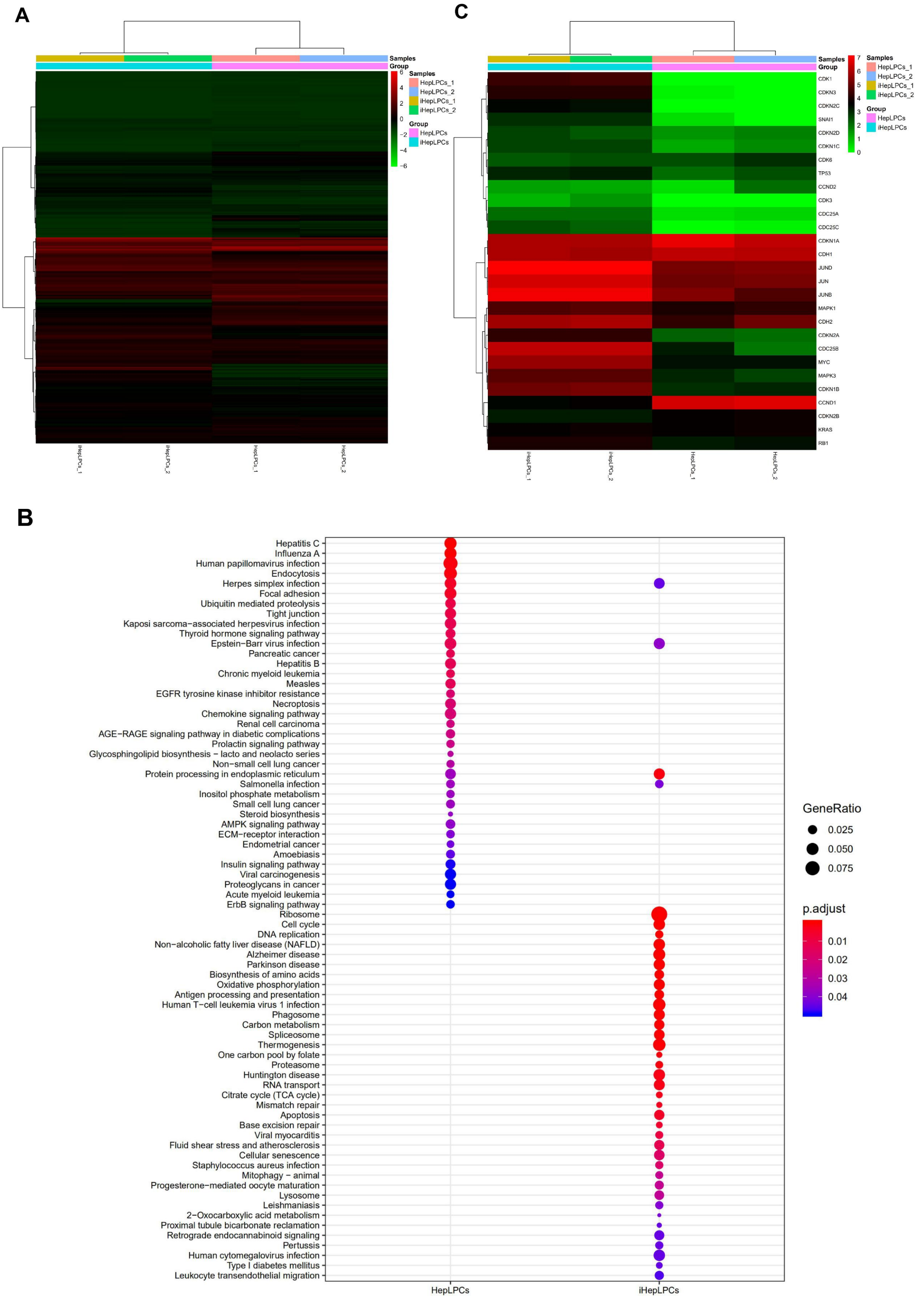
A
C
B
Samples
Group
HepLPCs_1
HepLPCs_2
iHepLPCs_1
iHepLPCs_2
HepLPCs
iHepLPCs
CDK1
CDKN3
CDKN2C
SNAI1
CDKN2D
CDKN1C
CDK6
TP53
CCND2
CDK3
CDC25A
CDC25C
CDKN1A
CDH1
JUND
JUN
JUNB
MAPK1
CDH2
CDKN2A
CDC25B
MYC
MAPK3
CDKN1B
CCND1
CDKN2B
KRAS
RB1
Hepatitis C
Influenza A
Human papillomavirus infection
Endocytosis
Herpes simplex infection
Focal adhesion
Ubiquitin mediated proteolysis
Tight junction
Kaposi sarcoma−associated herpesvirus infection
Thyroid hormone signaling pathway
Epstein−Barr virus infection
Pancreatic cancer
Hepatitis B
Chronic myeloid leukemia
Measles
EGFR tyrosine kinase inhibitor resistance
Necroptosis
Chemokine signaling pathway
Renal cell carcinoma
AGE−RAGE signaling pathway in diabetic complications
Prolactin signaling pathway
Glycosphingolipid biosynthesis − lacto and neolacto series
Non−small cell lung cancer
Protein processing in endoplasmic reticulum
Salmonella infection
Inositol phosphate metabolism
Small cell lung cancer
Steroid biosynthesis
AMPK signaling pathway
ECM−receptor interaction
Endometrial cancer
Amoebiasis
Insulin signaling pathway
Viral carcinogenesis
Proteoglycans in cancer
Acute myeloid leukemia
ErbB signaling pathway
Ribosome
Cell cycle
DNA replication
Non−alcoholic fatty liver disease (NAFLD)
Alzheimer disease
Parkinson disease
Biosynthesis of amino acids
Oxidative phosphorylation
Antigen processing and presentation
Human T−cell leukemia virus 1 infection
Phagosome
Carbon metabolism
Spliceosome
Thermogenesis
One carbon pool by folate
Proteasome
Huntington disease
RNA transport
Citrate cycle (TCA cycle)
Mismatch repair
Apoptosis
Base excision repair
Viral myocarditis
Fluid shear stress and atherosclerosis
Cellular senescence
Staphylococcus aureus infection
Mitophagy − animal
Progesterone−mediated oocyte maturation
Lysosome
Leishmaniasis
2−Oxocarboxylic acid metabolism
Proximal tubule bicarbonate reclamation
Retrograde endocannabinoid signaling
Pertussis
Human cytomegalovirus infection
Type I diabetes mellitus
Leukocyte transendothelial migration
HepLPCs
iHepLPCs
GeneRatio
0.025
0.050
0.075
p.adjust
0.01
0.02
0.03
0.04

Figure S3: Transcriptomics analysis of HepLPCs and iHepLPCs. (A) A heat map representation of the whole-genome transcriptome of HepLPCs versus iHepLPCs. (B) KEGG pathway enrichment analysis of the differentially expressed genes. (C) A heat map showing the expression of 28 proliferation-related genes in HepLPCs versus iHepLPCs. Each element represents log2 ($P<0.05$), as scaled by the corresponding color legends from 2 donors.

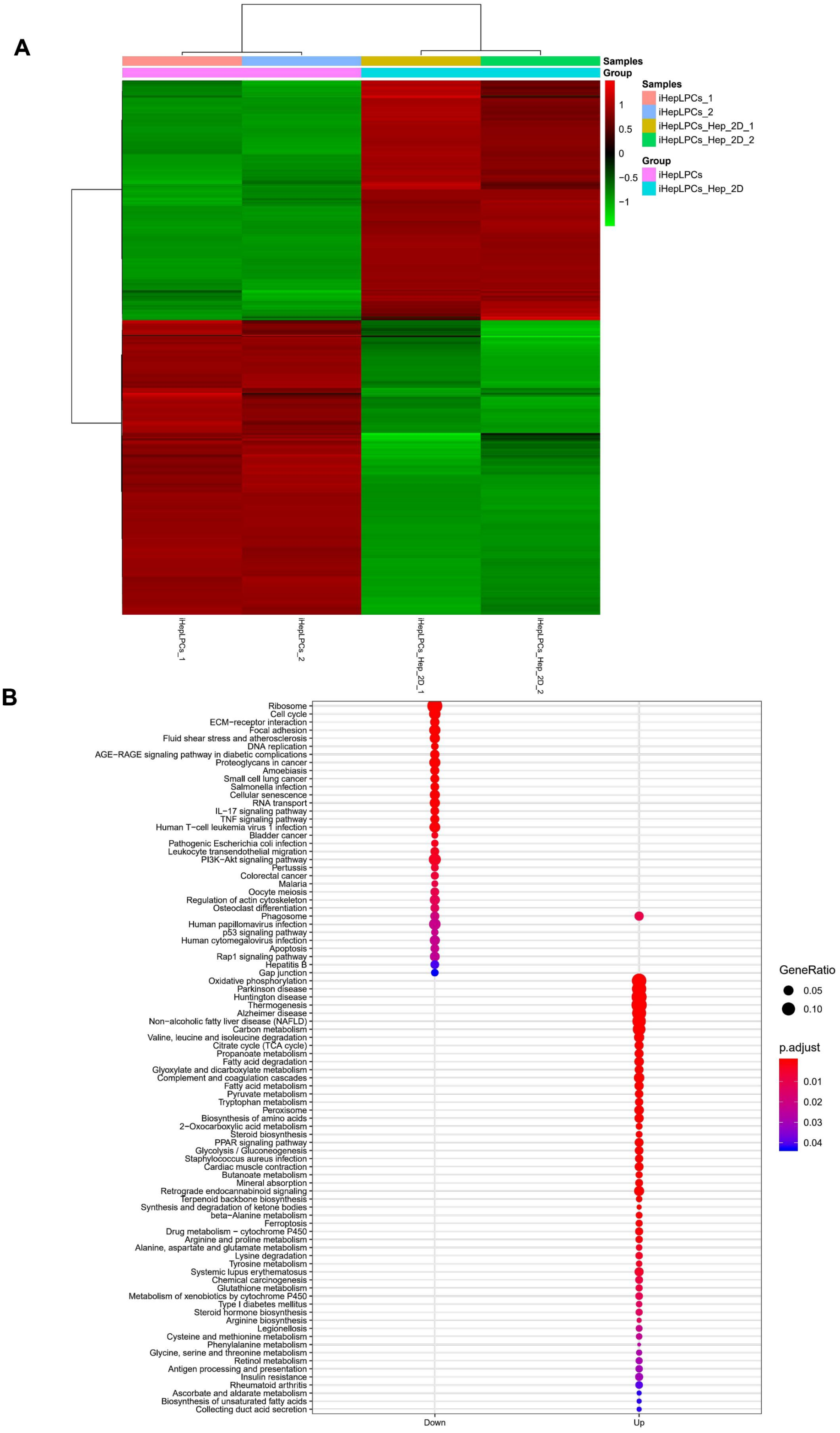

Figure S4: Transcriptomics analysis of iHepLPCs and iHepLPCs-Hep. (A) Euclidean hierarchical clustering of iHepLPCs versus iHepLPCs-Hep using differentially expressed genes (≥2-fold changes and $P < 0.05$) and (B) KEGG pathway enrichment analysis of the corresponding region.

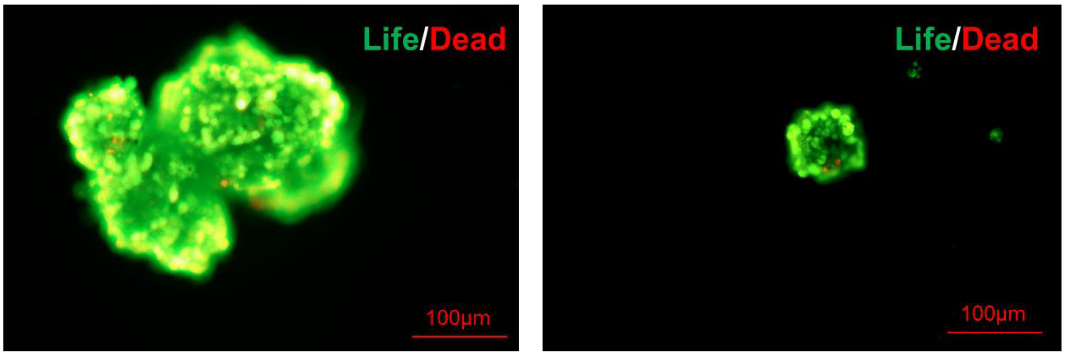

Figure S5: Life/Dead staining images. Green: Live cells; red: Dead cells. The results showed that the majority of cells were viable on the 9th day. Only a few of dead cells were randomly present in the spheroids, even in those which diameter is greater than 200μm

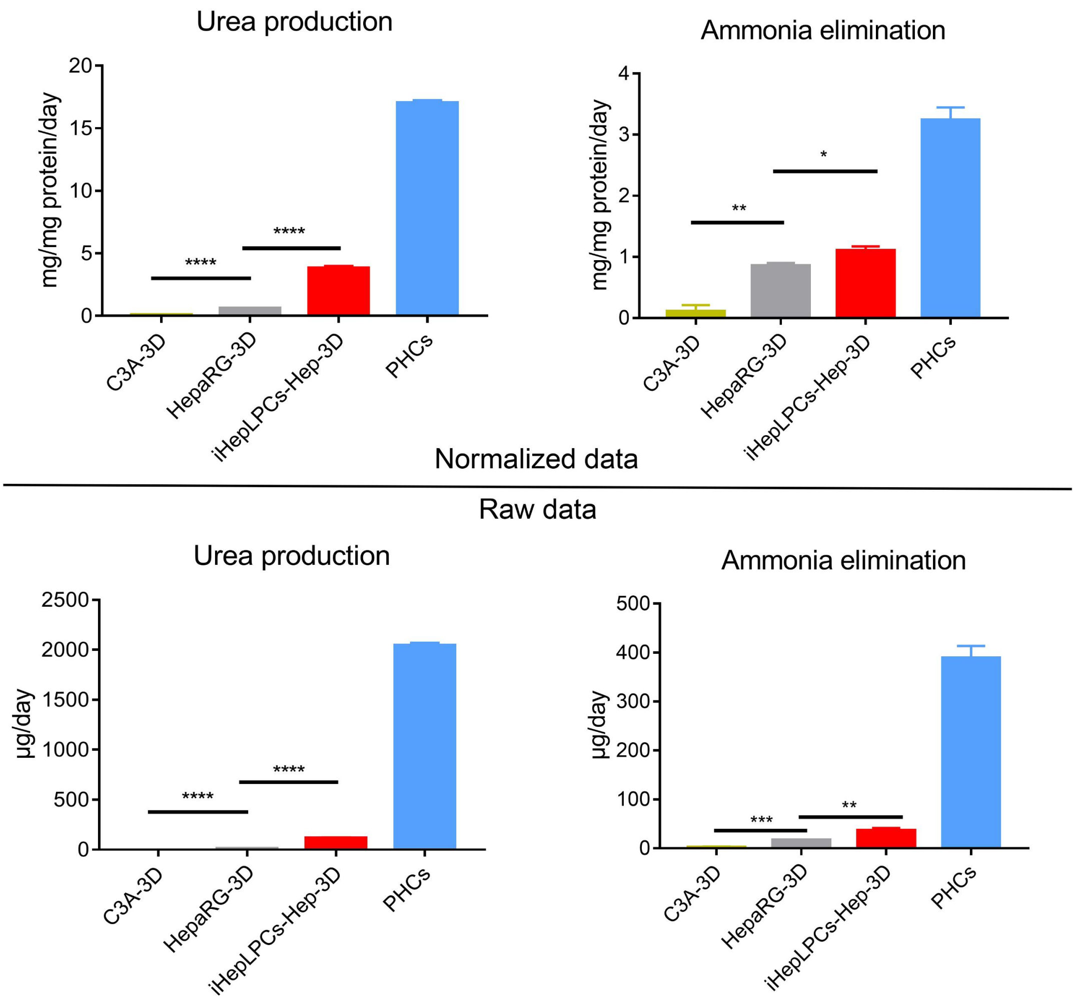

Figure S6: Urea production and ammonia elimination in C3A-3D, HepaRG-3D, and iHepLPCs-Hep-3D, compared to freshly isolated primary hepatocytes. iHepLPCs-Hep-3D produced a higher level of urea and eliminated ammonia more efficiently than other cell lines commonly used in a bioartificial liver. Error bars represent s.d.; *P < 0.05, **P < 0.01, ***P<0.001, ****P<0.0001; n = 3.

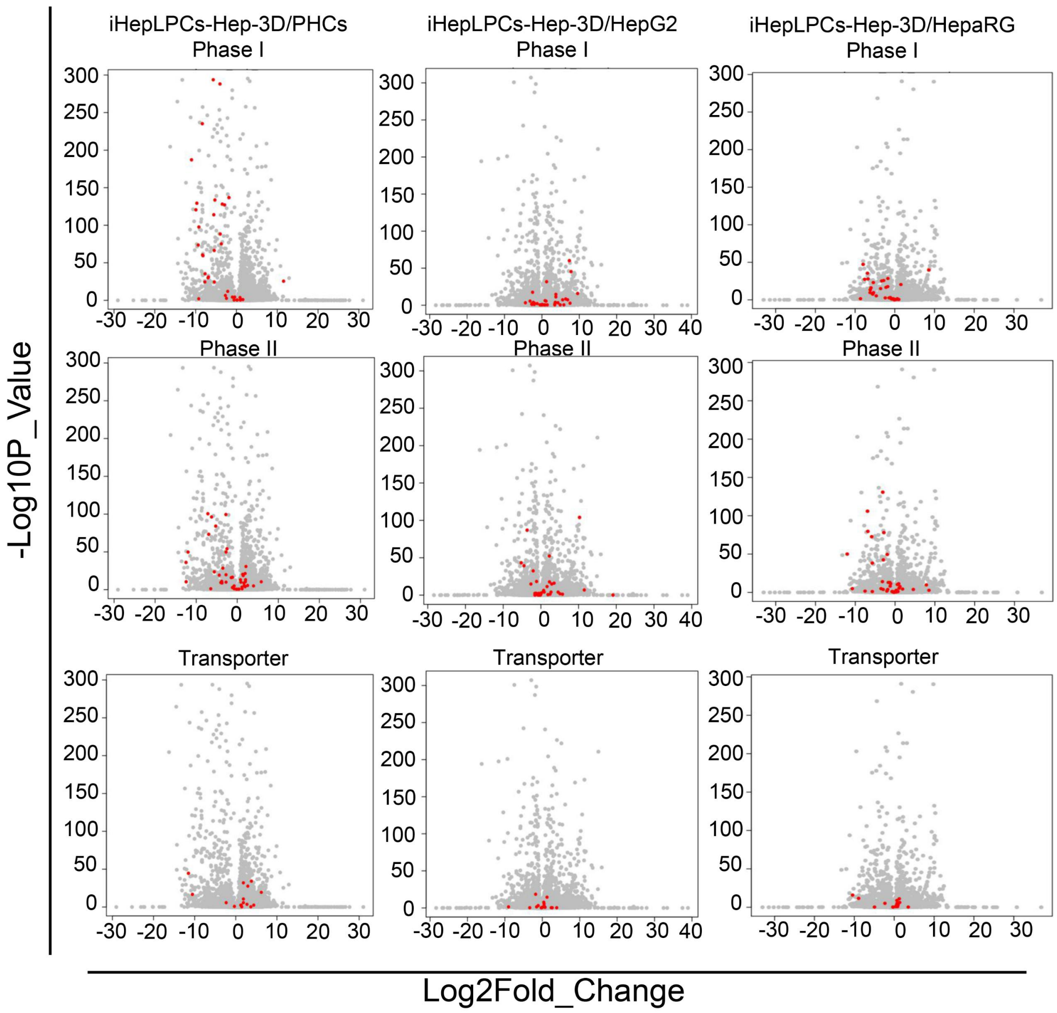

Figure S7: Volcano plot analysis of the expression of drug metabolizing enzymes and transport (DMET) genes in HepG2, HepaRG, and iHepLPCs-Hep-3D compared with PHCs. Genes with log2 fold changes for 36 phase I drug metabolizing enzymes, 46 major phase II drug metabolizing enzymes and 25 phase III transporter are shown by volcano plots.

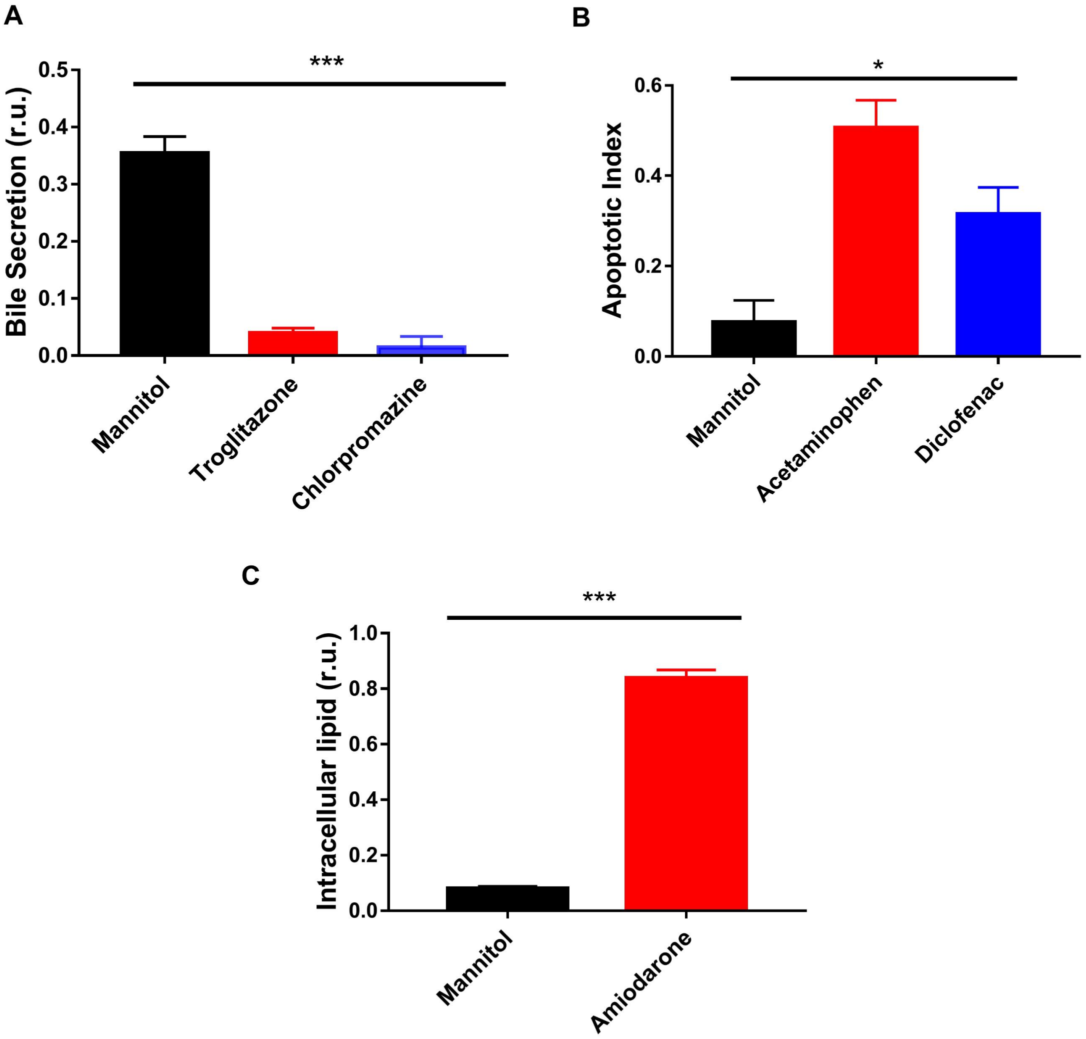

Figure S8: Quantitative analysis of fluorescence analysis of adverse outcome pathway in iHepLPCs-Hep-3D. Loss of bile acid production (cholestasis) evaluated by CDFDA staining, lipid accumulation (steatosis) by Nile Red staining and apoptosis by TUNEL labeling of nuclei. (A) Cholestasis in iHepLPCs-Hep-3D exposed to Troglitazone, Chlorpromazine or Mannitol (negative control) for 48 h (***P < 0.001; n = 3). (B) Apoptosis of differentiated hepatocytes following 48 h of exposure to Acetaminophen, Diclofenac or Mannitol (*P < 0.05; n = 3). (F) Steatosis in iHepLPCs-Hep-3D after 48 h of exposure to Amiodarone or Mannitol (***P < 0.001; n = 3). All error bars indicate ± s.d.

A

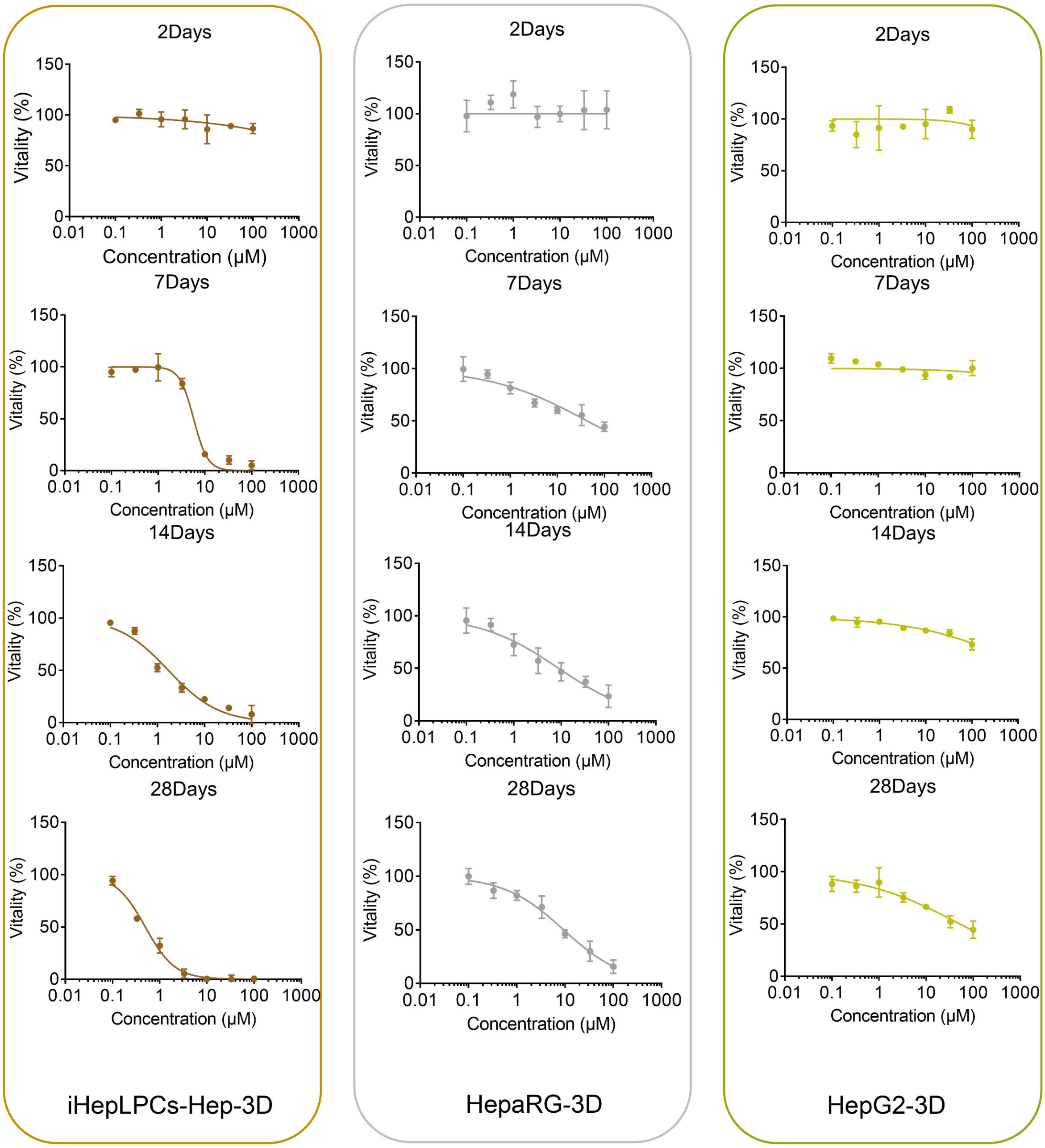

B

	TC_{50} of 2 days (μM)	TC_{50} of 7 days (μM)	TC_{50} of 14 days (μM)	TC_{50} of 28 days (μM)
**iHepLPCs-Hep-3D**	**n.d.**	**5.8±0.2**	**1.7±0.1**	**0.5±0.1**
**HepaRG-3D**	**n.d.**	**39.7±3.4**	**10.6±5.9**	**9.3±0.3**
**HepG2-3D**	**n.d.**	**n.d.**	**n.d.**	**55±8.2**

Figure S9: Long-term toxicological outcomes of iHepLPCs-Hep-3D. (A) Dose-dependent toxicity curves of fialuridine at the indicated time in iHepLPCs-Hep-3D, HepaRG-3D, and hepG2-3D. (B) TC_{50} values of fialuridine at the indicated time in iHepLPCs-Hep-3D, HepaRG-3D and hepG2-3D; n.d. not determined.

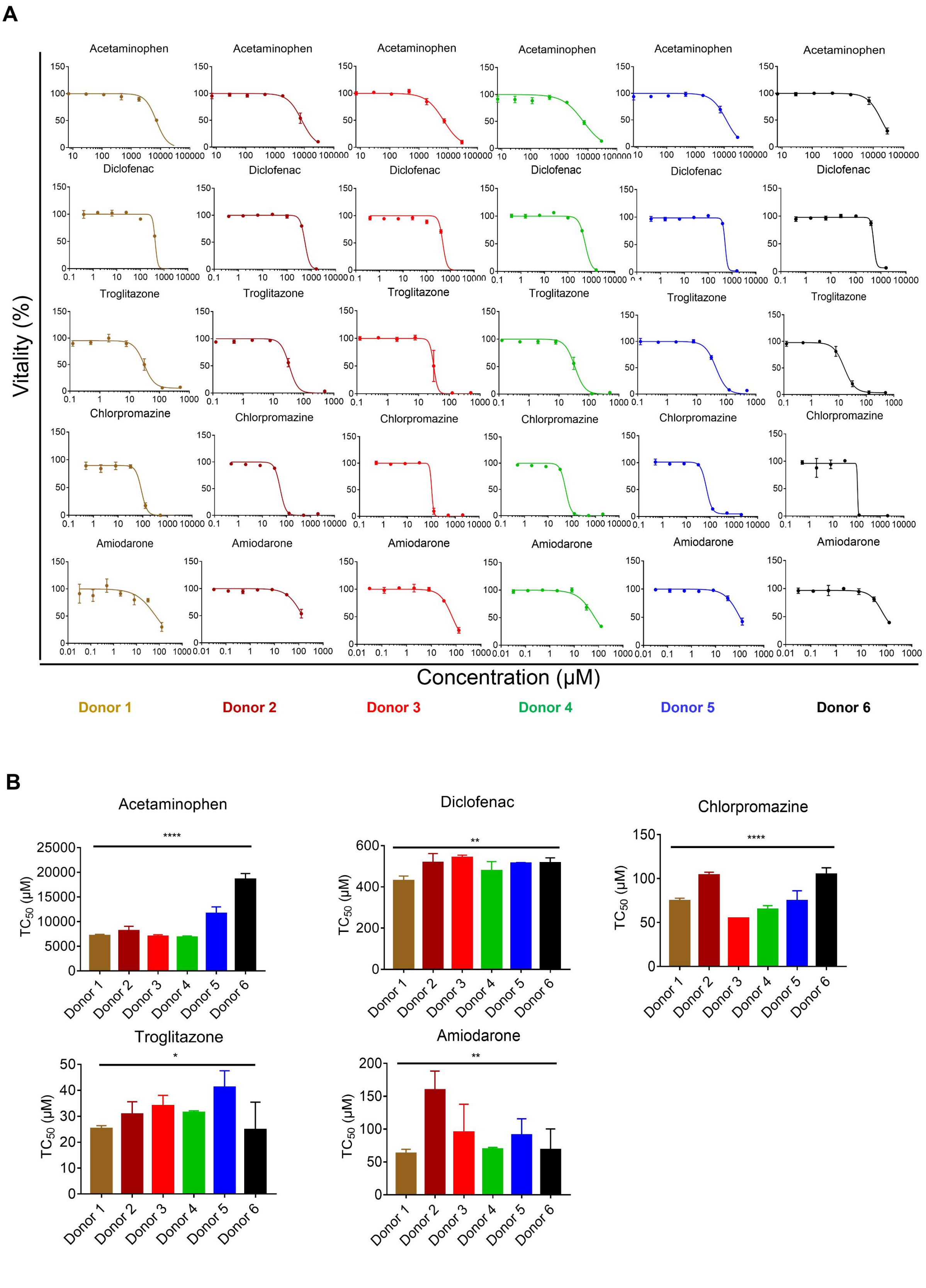
A
Acetaminophen
Diclofenac
Troglitazone
Chlorpromazine
Amiodarone
Vitality (%)
Concentration (µM)
Donor 1
Donor 2
Donor 3
Donor 4
Donor 5
Donor 6
B
Acetaminophen
****
Diclofenac
**
Chlorpromazine
****
Troglitazone
*
Amiodarone
**
TC50 (µM)

Figure S10: iHepLPCs-Hep-3D from different donors demonstrated significant individual heterogeneity. (A) Dose-dependent toxicity curves of different compounds obtained from 48-h dose responses in iHepLPCs-Hep-3D derived from different donors. (B) One-way ANOVA with Tukey correction for multiple comparisons showed that there was significant difference in the TC_{50} of iHepLPCs-Hep-3D from different donors; *$P < 0.05$, **$P < 0.01$, ****$P<0.0001$; $n = 3$; error bars indicate ± s.d.

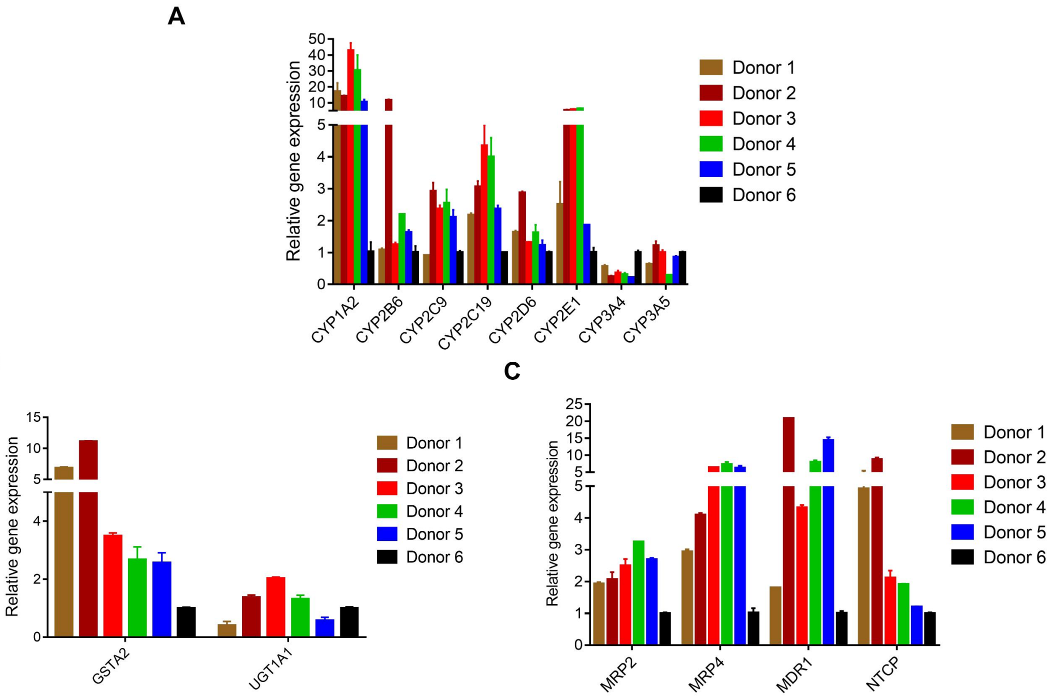

Figure S11: Heterogeneity in the expression of drug metabolism genes in 3D-iHepLPC-Heps derived from different donors. qPCR analyses showed that there was a significant heterogeneity in the expression of (A) eight phase I enzymes, (B) two phase II enzymes, and (C) four transporters. Error bars indicate ± s.d; n=3.

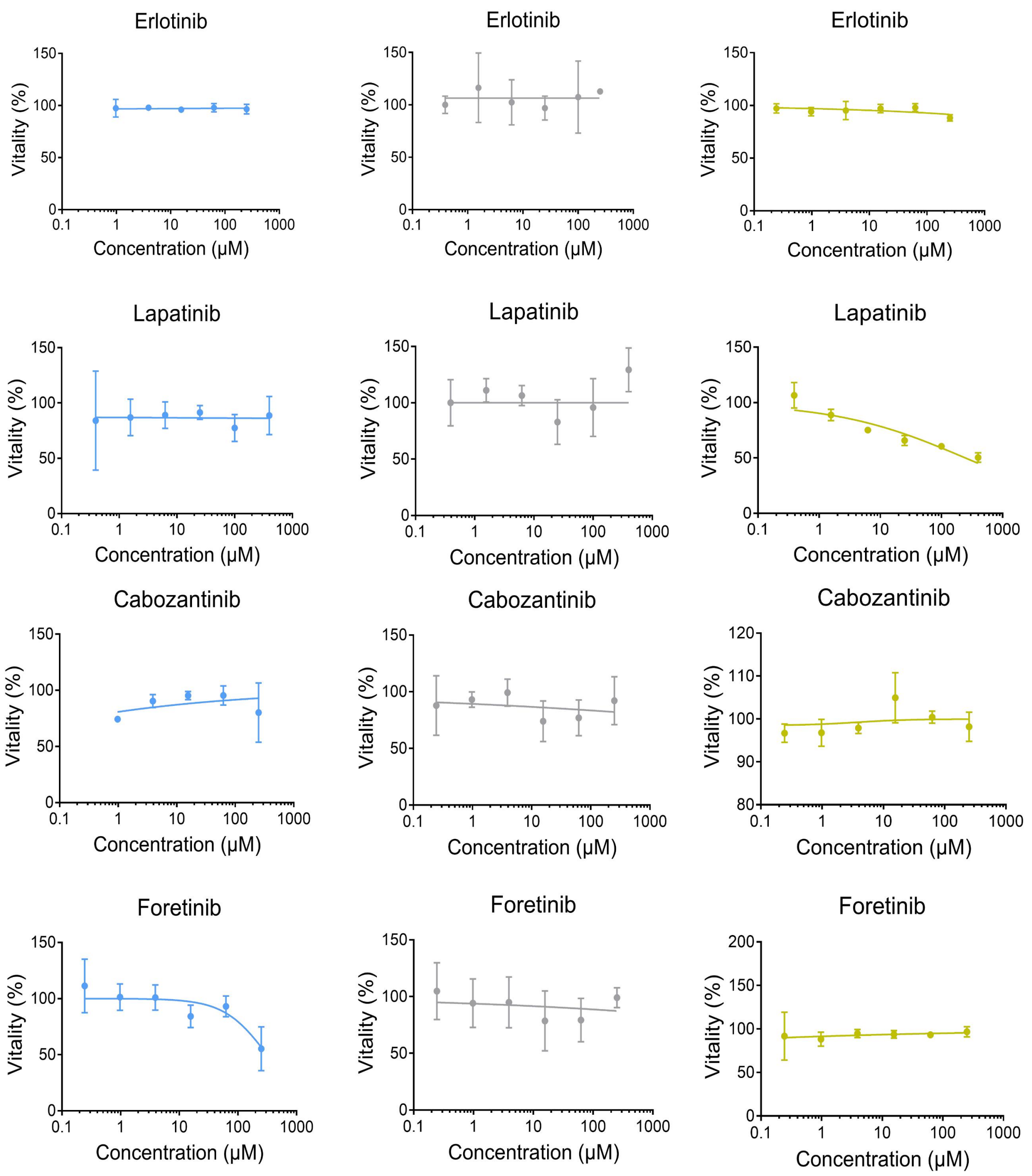

Figure S12: Dose-dependence curves of four new drugs, Erlotinib, Lapatinib, Cabozantinib, and Foretinib, in PHCs-3D, HepaRG-3D, and HepG2-3D. Apart from Lapatinib (with significant toxicity on HepG2-3D) and Foretinib (had a mild toxic reaction on PHCs-3D), no toxic reaction was found on HepaRG-3D, HepG2-3D, and PHCs-3D.

Gene	Forward sequence 5' -> 3'	Reverse sequence 5' -> 3'
18s	CAGCCACCCGAGATTGAGCA	TAGTAGCGACGGGCGGTGTG
ALB	GAGACCAGAGGTTGATGTGATG	AGTTCCGGGGCATAAAAGTAAG
CYP1A2	CTGGGCACTTCGACCCTTAC	TCTCATCGCTACTCTCAGGGA
CYP2A6	TTCAATCCCCAGCACTTCCT	GAAGTTCTGCATGACGGTGG
CYP3A5	GGTGGTGATTCCAACTTATGCT	GCGTGTCTAATTTCAAGGGGA
CYP2E1	ATGTCTGCCCTCGGAGTCA	CGATGATGGGAAGCGGGAAA
CYP3A4	AAGTCGCCTCGAAGATACACA	AAGGAGAGAACACTGCTCGTG
CYP2C9	GCCTGAAACCCATAGTGGTG	GGGGCTGCTCAAAATCTTGATG
CYP2C19/CYP2C	GGAAAACGGATTTGTGTGGGA	GGTCCTTTGGGTCAATCAGAGA
CYP2D6	TGGCAAGGTCCTACGCTTC	GCCACCACTATGCACAGGTT
CYP2B6	CCGGGGATATGGTGTGATCTT	CCGAAGTCCCTCATAGTGGTC
PXR	TTGCCCATCGAGGACCAGAT	GTCTCCGCGTTGAACACTGT
FXR	TGCAGATCAGACCGTGAATGA	TTGGTTGCCATTTCCGTCAAA
CAR	GATGCTGGCATGAGGAAAGAC	TTGCTCCTTACTCAGTTGCAC
MDR1	GGGAGCTTAACACCCGACTTA	GCCAAAATCACAAGGGTTAGCTT
BSEP	TTGGCTGATGTTTGTGGGAAG	CCAAAAATGAGTAGCACGCCT
UGT1A1	CTGTCTCTGCCCACTGTATTCT	TCTGTGAAAAGGCAATGAGCAT
RXRA	GGAGGTGAGGGAGGAGTT	GCATGAGTTAGTCGCAGACAT
NTCP	TGCTCTTCCCCACATTGATG	TCCTGGTTCTCATTCCTTGC
GSTA2	TACTCCAATATACGGGGCAGAA	TCCTCAGGTTGACTAAAGGGC
a-AT	GATCAACGATTACGTGGAGAAGG	CCTAAACGCTTCATCATAGGCA
apoB	TGCTCCACTCACTTTACCGTC	TAGCGTCCAGTGTGTACTGAC
CPS1	AATGAGGTGGGCTTAAAGCAAG	AGTTCCACTCCACAGTTCAGA
ARG1	GTGGAAACTTGCATGGACAAC	AATCCTGGCACATCGGGAATC
MRP2	GATTGCAGAGTCGCTTGAGG	GGTTGTTGCATTCGGTTCCT

Table S1：Primer list.

	Doubling-time（h）					
Generations	Donor1	Donor2	Donor3	Donor4	Donor5	Donor6
10	27.62±0.3	29.41±1.4	30.43±0.7	28.92±0.1	30.61±1.1	30.13±0.3
20	29.30±0.4	26.19±0.5	29.67±0.5	30.90±0.3	30.25±0.6	29.60±0.4
30	28.26±0.3	29.30±0.4	30.66±0.6	28.32±0.1	29.59±0.5	29.40±0.3
40	32.06±0.5	29.94±0.2	32.03±0.5	29.65±0.4	30.25±0.8	29.02±0.7

Table S2: Doubling time of different generations of iHepLPCs from six different donors.

Compound	Target	Main Indications	Cases of Serum Alanine Aminotransferase Elevation	Cases of Severe Hepatotoxicity (Death)	Reference
Foretinib	c-MET	HCC	Yes	No (no)	[1]
Cabozantinib	VEGFR,c-MET	Medullary thyroid CA	Yes	No (no)	[2, 3]
Tepotinib	c-MET	HCC	Yes	No (no)	NCT01988493
Crizotinib	c-MET	NSCLC	Yes	Yes (yes)	[2, 3]
Lapatinib	EGFR, ErbB2	Breast CA	Yes	Yes (yes)	[2, 3]
Gefitinib	EGFR, ErbB2	NSCLC	Yes	Yes (no)	[2, 3]
Erlotinib	EGFR, ErbB2	NSCLC, pancreatic CA	Yes	Yes (no)	[2, 3]
Ceritinib	EGFR, ErbB2	NSCLC , Anaplastic Lymphoma	Yes	Yes (no)	NCT01685060 NCT01283516
EGFR: Epidermal growth factor receptor; VEGFR: Vascular endothelial growth factor receptor; HCC: Hepatic cell carcinoma; NSCLC: Non-small-cell lung cancer; CA: Cancer.					

Table S3: Molecular targeted drugs tested in this study.

	HepG2	HepaRG	PHCs	iHepLPCs
**In vitro proliferation capacity**	Unlimited proliferation	Unlimited proliferation	Low	High
**Functional activities**	Low functional activities	High functional activities but need 2%DMSO and time consuming	High functional activities but rapid loss of function	Slightly lower than HepaRG' functional activities but rapid differentiati on
**Ammonia detoxification capacity**	Low (C3A cell line)	Slightly lower than iHepLPCs	High	High
**Suitable for bioartificial liver or not**	Not suitable	Not suitable	Not suitable	suitable
**Drug toxicity screening**	Inaccurate	Accurate but unpredict able to predict idiosyncratic drug induced liver injury	The gold standard	Accurate and can predict idiosyncratic drug induced liver injury

Table S4: Summary of main conclusions.

Supplement References

1. Peters S, Adjei AA. MET: a promising anticancer therapeutic target. Nature reviews Clinical oncology. 2012; 9: 314-26.
2. Bunchorntavakul C, Reddy KR. Drug Hepatotoxicity: Newer Agents. Clin Liver Dis. 2017; 21: 115-34.
3. Liu S, Kurzrock R. Toxicity of targeted therapy: Implications for response and impact of genetic polymorphisms. Cancer Treat Rev. 2014; 40: 883-91.
